# Association of Serum miR-186-5p With the Prognosis of Acute Coronary Syndrome Patients After Percutaneous Coronary Intervention

**DOI:** 10.3389/fphys.2019.00686

**Published:** 2019-06-05

**Authors:** Zhuoling Li, Jia Wu, Weishi Wei, Xiaomin Cai, Jing Yan, Jiaxi Song, Cheng Wang, Junjun Wang

**Affiliations:** ^1^Department of Clinical Laboratory, Jinling Hospital, School of Medical, Nanjing University, Nanjing, China; ^2^School of Medicine, Jiangsu University, Zhenjiang, China; ^3^Department of Cardiology, Jinling Hospital, School of Medical, Nanjing University, Nanjing, China

**Keywords:** microRNA, biomarker, acute coronary syndrome, percutaneous coronary intervention, major adverse cardiovascular event

## Abstract

Circulating miR-186-5p is an emerging biomarker for acute coronary syndrome (ACS) patients. However, its kinetic signatures and prognostic values in ACS patients undergoing percutaneous coronary intervention (PCI) remain unclear. Levels of serum miR-186-5p were determined in 96 healthy controls and 92 ACS patients before and after PCI by qRT-PCR, and the physiologic state of miR-186-5p was analyzed by comparing its absolute concentrations in isolated exosomes and exosome-depleted supernatants. An average of 1 year of follow-up for ACS patients after PCI was performed. MiR-186-5p levels in the myocardium and serum of rats following left anterior descending coronary artery (LAD) ligation were measured. Serum miR-186-5p levels were found to be significantly increased in ACS patients upon admission compared with those of controls, but these high miR-186-5p levels gradually decreased within 1 week after PCI. Serum miR-186-5p was mainly present in an exosome-free form rather than membrane-bound exosomes. Within 1 year of follow-up, ACS patients with higher miR-186-5p levels upon admission exhibited a higher incidence of MACE after PCI. Different statistical analyzes further validated the independent prognostic values of serum miR-186-5p in ACS patients after PCI. Serum miR-186-5p levels in rats following LAD ligation were increased, and there was a decrease in myocardial miR-186-5p levels. Kyoto encyclopedia of genes and genomes (KEGG) analysis was performed to predict the related pathways of target genes of miR-186-5p, which suggested that miR-186-5p might be involved in ACS by regulating the inflammatory status and D-glucose metabolism. In conclusion, a distinctive expression signature of serum miR-186-5p may contribute to monitoring the clinical condition and assessing the prognosis of ACS patients undergoing PCI.

## Introduction

Ischemic heart disease (IHD) is one of the leading causes of morbidity and mortality worldwide, and ischemic heart disease has caused the most global deaths in the last few decades (GBD 2016 Causes of Death Collaborators, 2017). Acute coronary syndrome (ACS), the main form of IHD and the most serious medical emergency, occurs as a result of myocardial ischemia after unstable coronary atherosclerotic plaque formation, and its clinical presentations include acute myocardial infarction (AMI) and unstable angina (UA) (Carreras and Mega, 2015). Currently, percutaneous coronary intervention (PCI) is the mainstay of treatment for ACS to restore coronary perfusion and reduce infarct size during acute myocardial ischemia (O’Gara et al., 2013). Nevertheless, ACS patients are prone to recurrent angina and coronary restenosis after PCI due to patients’ predisposing factors, such as myocardial insult, inflammatory disorders, and thrombogenic tendencies (Tsai et al., 2017; Anroedh et al., 2018; Sardu et al., 2018). There is still a lack of highly sensitive and specific approaches to monitor the clinical condition and assess the prognosis of ACS patients. This current situation underscores the need for further exploration of reliable biomarkers that could reflect improvement of cardiac function in ACS patients after reperfusion therapy.

Circulating microRNAs (miRNAs) have emerged as promising markers of cardiovascular diseases and are closely associated with the pathogenesis of atherosclerosis and ischemic heart disease (Economou et al., 2015). miRNAs can be actively or passively released as intercellular communication mediators and subsequently serve as non-invasive biomarkers for assessing disease progression (Chen et al., 2017; de Couto et al., 2017). Accumulating studies have demonstrated that numerous circulating miRNAs markedly change before and after PCI treatment and could be used as the novel approach for clinical monitoring and prognostic evaluation of ACS patients (Chen et al., 2015; Yang et al., 2016; Zhu et al., 2016; Zhang et al., 2018). A few reports identified the favorable diagnostic performance of circulating miR-186-5p for predicting the presence of AMI or UA (Zeller et al., 2014; Liu X. et al., 2016). Our previous study also revealed that serum miR-186-5p levels exhibited a more significant increase in ACS patients than that in stable CAD patients, contributing to the comprehensive risk stratification of atherosclerotic cardiovascular diseases (Wu et al., 2014). However, the kinetic signatures of circulating miR-186-5p in ACS patients after reperfusion therapy remain unclear, and their association with ACS prognosis still needs exhaustive evaluations. Furthermore, there has been no report on the potential source and predominant form of dysregulated circulating miR-186-5p in ACS patients. It would be interesting to explore the physiologic status of specific miRNAs in the blood circulation of ACS patients to help us gain a better understanding of their value for monitoring and prognosis.

In the present study, we analyzed the changes in serum miR-186-5p levels in ACS patients upon admission (before PCI) and at different time points after PCI treatment using quantitative reverse-transcription PCR (qRT-PCR). To investigate the potential source of serum miR-186-5p, we compared the differences in miR-186-5p levels between serum and myocardial tissues of rats following permanent ligation of the left anterior descending (LAD) coronary artery. To further explore the predominant form of serum miR-186-5p, we isolated exosomes from serum samples and compared the differences in miR-186-5p concentrations between exosomes and corresponding exosome-depleted supernatants. To assess the association of serum miR-186-5p with ACS prognosis after PCI treatment, we performed an average of one-year follow-up evaluation and calculated the incidence of major adverse cardiovascular events (MACE) in ACS patients. To preliminarily explore the miR-186-5p related pathways in ACS progress, we predict target genes of miR-186-5p by using bioinformatics approaches and performed Kyoto Encyclopedia of Genes and Genomes (KEGG) analysis.

## Materials and Methods

### Study Subjects and Blood Sampling

From January 2017 to September 2017, 92 consecutive eligible patients with ACS and 96 control subjects were enrolled. All subjects gave written informed consent in accordance with the Declaration of Helsinki. This study was approved by the Research Ethics Committee of Jinling Hospital (2015NZGKJ-018) and was performed in accordance with the Declaration of Helsinki of 1975, as revised in 2013.

All ACS patients were newly diagnosed and underwent PCI for the first time. The diagnosis of AMI was according to the universal definition of AMI (Thygesen et al., 2012) and was defined as ischemic chest pain lasting >30 min, cardiac biomarker elevation [cardiac troponin (cTnI) >0.06 ng/mL and/or creatine kinase MB (CK-MB) > 16 IU/L], and electrocardiogram (ECG) showing a new ST-segment shift in two or more contiguous leads. The diagnosis of unstable angina pectoris was defined as patients with clinical history and ECG consistent with ACS without elevation of cardiac biomarkers. Patients associated with a history of coronary artery bypass grafting surgery, myocardial infarction, cardiogenic shock, stroke, malignant diseases and various acute or chronic infectious diseases were excluded.

Ninety-six age- and gender-matched control volunteers without any other definitive diseases such as hypertension, hyperlipidemia, or cardiovascular and cerebrovascular diseases, were recruited contemporaneously from the Healthy Physical Examination Center of Jinling Hospital.

Peripheral venous blood samples of ACS patients were collected upon admission (before PCI) and within 1 day (≤24 h), 1∼2 days (24∼48 h), 2∼3 days (48∼72 h), and 4 ∼7 days after PCI. Fasting blood samples of the controls were also collected. All samples were immediately centrifuged at 3500 rpm for 5 min at room temperature, and the obtained serum supernatants were stored at −80°C until RNA extraction.

### Clinical Scores and Follow-Ups

Gensini scores were used to assess the severity of coronary artery stenosis (Sinning et al., 2013). Global Registry of Acute Coronary Events (Grace) scores were calculated (by the Grace 2.0 calculator online^1^) within 3 days after hospitalization (before PCI) and before discharge (after PCI) to predict the clinical outcome of ACS patients. Traditional echocardiographic parameters, such as LV endsystolic volume, left ventricular ejection fraction (LVEF), and LV mass index, were obtained from 2D, M-mode from three consecutive cardiac circles by Vivid seven color Doppler imaging (General Electric Company, Co., United States) (Tao et al., 2015). wall motion of 17 segments was visually assessed by consensus-reading by one experienced physician and WMSI (wall motion score index) was calculated as the sum of the segment scores divided by the number of segments score (1 = normokinesis, 2 = hypokinesis, 3 = akinesis, 4 = dyskinesis) (Kumarathurai et al., 2016). An average of one-year follow-up was made either by outpatient visit or by telephonic interview. The primary end point was the occurrence of MACE, which was defined as a composite of all-cause mortality, non-fatal myocardial infarction (non-fatal MI), stroke and unplanned coronary revascularization.

### PCI Procedure

Percutaneous coronary intervention was performed according to the standard guidelines (Smith et al., 2001) by two or more experienced doctors who were blinded to the study, and femoral coronary intervention was the preferred operating approach. Coronary artery stenoses of ≥50% luminal narrowing were considered significant. Stents were placed when the stenosis of infarct-related arteries was above 70%; meanwhile, the type and number of affected arteries and stenosis severity were visually collected by operators. After PCI, the residual stenosis presented less than 20% and thrombolysis in myocardial infarction flow grade was determined in at least two in the infarct-related arteries. Patients were placed in one or more second-generation drug-eluting stents. Before undergoing catheterization, patients received 300 mg of chewable aspirin, 600 mg of clopidogrel orally, atorvastatin 40 mg orally, and intravenously injected 50–100 IU/kg heparin during the PCI procedure. All patients were on dual antiplatelet therapy for at least 1 year after coronary artery stenting.

### Biochemical Determination

Clinical biochemical parameters including serum triglyceride (TG), total cholesterol (TC), low-density lipoprotein cholesterol (LDL-C), high-density lipoprotein cholesterol (HDL-C), and glucose (GLU) levels upon admission were measured by a Hitachi 7600 autoanalyzer using commercial reagents (Hitachi High-Technologies Corporation, Tokyo, Japan). Serum CK-MB and cTnI levels were detected by an AIA 2000 autoanalyzer using commercial reagents (TOSOH Corporation, Tokyo, Japan). Serum N-terminal-pro-B-type natriuretic peptide (NT-pro-BNP) levels were determined by using an E601 electrochemiluminescence automatic immunoassay analyzer using commercial reagents (Roche Corporation, Basel, Switzerland). D-Dimer (DD) was detected by a CS5100 automatic blood coagulation analyzer (Sysmex America Inc., Mundelein, United States). Blood cell counts were measured by an automated Sysmex HST201 Hematology System (Sysmex America Inc., Mundelein, United States). Neutrophil-to-lymphocyte ratio (NLR), platelet-to-lymphocyte ratio (PLR) and the systemic immune inflammation index (SII, defined as platelet^*^neutrophil/lymphocyte) were calculated by using baseline values of neutrophils, lymphocytes, and platelets in peripheral blood (Fest et al., 2018).

### Animals and Experimental Design

Male Sprague-Dawley (SD) rats (RRID:MGI:5651135) (8∼10 weeks old) were obtained from the Model Animal Research Center of Jinling Hospital (MARC, Nanjing, China) and were housed in the professional facilities of the experimental animal resource. This study was carried out in accordance with the guidelines of the Institutional Animal Care and Use Committee of Jinling Hospital. The protocol was approved by the Institutional Animal Care and Use Committee of Jinling Hospital. AMI was induced by permanent LAD coronary artery ligation and the detail procedure was established as previously described (Yang et al., 2007; Liu X. et al., 2016). A total of 25 rats were randomly assigned to five groups: (1) the sham-operated group (*n* = 5): the same experimental open-chest surgery procedure was performed without LAD ligation; (2) the AMI-2h group: sacrificed at 2 h after LAD ligation (*n* = 5), (3) the AMI-4h group: sacrificed at 4 h after LAD ligation (*n* = 5), (4) the AMI-8h group: sacrificed at 8 h after LAD ligation (*n* = 5); (5) the AMI-24h group: sacrificed at 24 h after LAD ligation (*n* = 5). 3 mL celiac venous blood samples and infarcted left ventricular tissues were harvested from each rat before sacrifice and then centrifuged at 3500 rpm for 5 min at room temperature to obtain serum supernatants.

### Serum Exosome Isolation and Characterization

A commercial kit (Life Technologies, CA, United States) was used to isolate total exosomes from 100 μL of individual serum samples according to the manufacturer’s protocol. The resulting exosomes were suspended in 20 μL of PBS for miRNA analysis, and characterized by nanoparticle tracking analysis (NTA), transmission electron microscopy (TEM), and Western blot. After exosome isolation, the exosome-depleted supernatant was also collected and used for further RNA isolation.

A ZetaView® PMX 110 V3.0 (Particle Metrix, Meerbusch, Germany) was used to evaluate the size and concentration of exosomes. Isolated exosome pellets were diluted 1:2000 in PBS and 1 mL of the sample was loaded into the chamber. The size distribution profiles were analyzed by Software ZetaView 8.04.02 software. The morphology of exosomes was identified by using a Tecnai G2 20 (Hitachi, Tokyo, Japan). Resuspended exosome liquid was added on a 200 mesh copper grid and stained with 2% phosphotungstic acid (Sigma-Aldrich, Louis, MO, United States) 10 min later. The dried grids were examined at an acceleration voltage of 80 kV. Exosomal markers CD63, Alix and TSG 101 were determined by using Western blotting as previously described (Ding et al., 2018). 40 μg of protein from each sample was electrophoresed on a 10% SDS-PAGE gel and transferred onto a polyvinyl difluoride fluoride membrane. After blocking with 5% skim milk (w/v), membranes were blotted by primary antibodies against CD63 (Absin Bioscience, Cat# abs110250), Alix (Abcam, Cat# ab186429, RRID: AB_2754981), and TSG 101 (Abcam, Cat# ab83, RRID:AB_306450) at 4°C overnight, then, membranes were incubated with a secondary IgG-HRP (Santa Cruz Biotechnology Cat# sc-2004, RRID: AB_631746). Immunoreactive bands were visualized with an enhanced chemiluminescence detection kit (Thermo Fisher Scientific, MA, United States).

### RNA Extraction

First, total RNA from the serum of patients and rats was extracted from 100 μL of individual serum samples by a 1-step phenol/chloroform purification method as previously described (Wang et al., 2016). RNAs from exosomes, exosome-depleted supernatants and myocardial tissues were extracted and purified using Trizol Reagent (Invitrogen, CA, United States) following the manufacturer’s protocol. 2 μL of total RNA was reverse transcribed into complimentary DNA in a reaction volume of 10 μl with an AMV reverse transcriptase system (Takara, Otsu, Japan) by using TaqMan primer sets (Applied Biosystems, CA, United States) for miRNAs, as previously described (Lu et al., 2017).

### qRT-PCR Assay

qRT-PCR was performed on a 7300 Sequence Detection System by using TaqMan miRNA probes (Applied Biosystems, Foster City, CA, United States) according to the manufacturer’s protocols. All reactions were conducted in triplicate. To normalize the experimental qRT-PCR data, a synthetic MIR2911 (5′-GGCCGGGGGACGGGCUGGGA-3′) RNA oligonucleotide (10^6^ fmol/L) was applied as a spike-in control. MIR2911 has previously been reported to be conserved in plants and does not affect human miRNA detection, so it is widely used for RNA extraction efficiency normalization (Lu et al., 2017; Wan et al., 2017). Relative levels of serum miR-186-5p were normalized by the exogenous MIR2911 using the comparative Cq method (2^^–ΔCq^). ΔCq was calculated by subtracting the Cq values of MIR2911 from that of miR-186-5p. Since U6 is the most common endogenous control in the research for miRNA detection in tissues, relative levels of miR-186-5p in myocardial tissues were normalized by endogenous U6 using the same calculation method.

To effectively compare the absolute concentrations of miR-186-5p in total serum samples, their respective exosomes and exosome-depleted supernatants, we generated a standard curve of miR-186-5p with ten-fold serial dilutions of synthetic mature miR-186-5p oligonucleotide (TaKaRa, Dalian, China) from 10^2^ to 10^8^ fmol/L as previously described (Lu et al., 2017; Wan et al., 2017). The Cq values of the synthetic oligonucleotide were determined by qRT-PCR and were plotted versus the log10 of the concentrations of each calibrator.

### Target Gene Prediction and KEGG Analysis

The target mRNAs of miR-186-5p were predicted by bioinformatics software, including TargetScan, miRTarBase and the miRDB database. KEGG analyses were performed to predict their potential functions and related pathways by using the DAVID database^2^.

### Statistical Analysis

All statistical analyses were performed with SPSS 19.0 software (Chicago, IL, United States). The one-sample Kolmogorov-Smirnov Test was utilized to determine the normality of data distribution. Normal variables were presented as the mean ± SD, and r comparisons of normal variables between groups were analyzed by independent sample *t*-test. Data with abnormal distribution were expressed as the median [interquartile range, (IQR)], and their comparisons between groups were analyzed by Mann-Whitney *U* test. Categorical variables are presented as counts or percentages, and their comparisons between groups are analyzed by Chi-Square test. A paired *t*-test was used to compare paired data when the difference between groups was normally distributed; otherwise, the Wilcoxon signed-rank test was used. The correlation of serum miR-186-5p and clinical biochemical parameters was evaluated by Spearman’s rank correlation analysis. The predictive values of serum miR-186-5p were assessed by receiver-operating characteristic (ROC) curve and logistic regression analyses. Kaplan-Meier survival curves were constructed and compared using the log-rank test. Univariate and multivariate Cox regression analyses were applied to estimate independent prognostic factors. A two-tailed *p*-value less than 0.05 was considered statistically significant.

## Results

### Demographic and Baseline Characteristics of Study Populations

A total of 188 subjects, including 92 ACS patients (including 62 STEMI patients, 20 NSTEMI patients and 10 UA patients) and 96 healthy controls were enrolled in this study. Their baseline clinical and biochemical characteristics are summarized in Table 1. There were no significant differences in age and sex distributions between STEMI, NSTEMI, UA patients and healthy controls. Compared with the control group, the STEMI and NSTEMI groups exhibited markedly elevated TG, and all three subgroups showed decreased HDL-C and ALB levels while increased NLR, PLR, and SII levels and higher percentages of subjects with hypertension, diabetes mellitus and dyslipidemia and tobacco use. The majority of ACS patients had 3- vessel disease (VD) (40 patients, 43.4%), and the LAD coronary artery was the most commonly affected artery (41.8%). The mean stenosis severities of stent-placed vessels and stent-unplaced vessels were 92.7 and 42.6% before PCI in ACS patients. There were no significant differences in the type and number of affected arteries among STEMI, NSTEMI, and UA patients. Distribution of segments of abnormal wall motion and angiographic data of ACS patients were summarized in Supplementary Tables S1, S2. ACS patients with diabetes were found at a higher proportions of 3-VD than others (χ^2^ = 7.861, *p* = 0.005).

**TABLE 1:** The baseline clinical and biochemical characteristics of all the study populations.

**Variables**	**ACS patients (*n* = 92)**			**Controls (*n* = 96)**	***p^a^***	***p*^b^**	***p*^c^**
	**STEMI (*n* = 62)**	**NSTEMI (*n* = 20)**	**UA (*n* = 10)**				
Age, years, mean (SD)	62.7(10.7)	65.0(13.0)	60.8(12.1)	63.3(12.1)	0.739	0.590	0.532
Sex, male, no (%)	54(87.1)	15(75.0)	9(90.0)	83(86.5)	0.908	0.198	0.753
SBP, mmHg, mean (SD)	125.7(25.8)	135.5(21.2)	142.1(21.1)	N/A	N/A	N/A	N/A
DBP, mmHg, mean (SD)	73.0(13.2)	78.4(13.1)	78.8(15.2)	N/A	N/A	N/A	N/A
TC, mmol/L, mean (SD)	4.41(0.97)	4.80(1.07)	4.36(0.73)	4.49(0.58)	0.540	0.097	0.611
TG, mmol/L, median (IQR)	1.54(1.04–2.08)	1.83(1.22–2.54)	1.10(0.76–1.62)	1.04(0.79–1.31)	<0.001	<0.001	0.700
HDL-C, mmol/L, median (IQR)	0.96(0.87–1.23)	1.05(0.95–1.35)	0.93(0.85–1.18)	1.38(1.19–1.56)	<0.001	0.023	0.005
LDL-C, mmol/L, mean (SD)	2.44(0.80)	2.69(0.66)	2.63(0.49)	2.69(0.58)	0.167	0.997	0.818
GLU, mmol/L, median (IQR)	8.25(6.33–11.38)	7.70(5.80–13.10)	6.35(4.53–8.33)	5.00(4.78–5.43)	<0.001	<0.001	0.087
NLR, median (IQR)	3.35(1.83–6.72)	5.14(3.13–6.16)	2.49(1.96–4.69)	1.74(1.35–2.23)	<0.001	<0.001	0.012
PLR, median (IQR)	124.7(78.9–198.6)	114.7(80.2–158.6)	117.1(91.6–226.8)	100.9(76.9–131.0)	0.002	0.048	0.041
SII, median (IQR)	662.7(376.7–1322.7)	910.9(597.3–1213.4)	559.0(297.5–1164.0)	331.5(253.0–473.8)	<0.001	0.001	0.044
ALB, g/L, median (IQR)	41.8(37.6–44.0)	39.8(37.7–44.1)	41.6(38.0–43.5)	44.3(43.6–48.1)	0.006	0.004	0.004
DD, mg/L, median (IQR)	0.34(0.19–0.50)	0.29(0.09–0.73)	0.38(0.25–0.56)	N/A	N/A	N/A	N/A
NT-pro-BNP, pmol/L, median (IQR)	21.8(5.80–113.40)	127.3(13.75–436.90)	33.50(30.90–60.40)	N/A	N/A	N/A	N/A
cTnI, ng/mL, median (IQR)	0.26(0.06–5.89)	0.13(0.04–6.43)	0.11(0.03–1.02)	N/A	N/A	N/A	N/A
CK, IU/L, median (IQR)	134.00(83.50–414.75)	106.00(43.25–346.25)	77.00(46.50–103.00)	N/A	N/A	N/A	N/A
CK-MB, IU/L, median (IQR)	18.00(9.00–61.00)	18.00(5.00–38.00)	10.00(4.00–19.00)	N/A	N/A	N/A	N/A
Hypertension (%)	45(72.6)	12(60.0)	9(90.0)	0(0)	<0.001	<0.001	<0.001
Diabetes mellitus (%)	24(38.7)	12(60.0)	4(40.0)	0(0)	<0.001	<0.001	<0.001
Dyslipipidemia (%)	17(27.4)	12(60.0)	4(40.0)	0(0)	<0.001	<0.001	<0.001
Tobacco use (%)	29(46.8)	13(65.0)	6(60.0)	23(24.0)	0.003	<0.001	0.015
Grace scores, mean (SD)	150.8(27.8)	138.0(37.8)	130.7(30.0)	N/A	N/A	N/A	N/A
Gensini scores, mean (SD)	66.6(30.9)	49.5(19.0)	72.2(27.7)	N/A	N/A	N/A	N/A
LVEF, %, mean,(SD)	55.3(8.1)	58.5(8.2)	60.9(7.3)	N/A	N/A	N/A	N/A
WMSI, mean, SD	1.19(0.22)	1.09(0.16)	1.10(0.17)	N/A	N/A	N/A	N/A
Number of affected vessel, no (%)							
1-VD	14(22.6)	2(10.0)	2(20.0)	N/A	N/A	N/A	N/A
2-VD	20(32.3)	11(55.0)	3(30.0)	N/A	N/A	N/A	N/A
3-VD	28(45.1)	7(35.0)	5(50.0)	N/A	N/A	N/A	N/A
Type of affected vessel, no (%)							
LM	3(4.8)	0(0)	0(0)	N/A	N/A	N/A	N/A
LAD	39(62.9)	19(95.0)	8(80.0)	N/A	N/A	N/A	N/A
LCX	26(41.9)	7(35.0)	7(70.0)	N/A	N/A	N/A	N/A
RCA	34(54.8)	8(40.0)	7(70.0)	N/A	N/A	N/A	N/A
Stenosis severity, mean, (SD)							
Overall vessels	71.6(12.1)	73.4(13.3)	81.8(15.1)	N/A	N/A	N/A	N/A
Stent-placed vessels	92.9(7.52)	92.4(7.09)	92.2(6.25)	N/A	N/A	N/A	N/A
Stent-unplaced vessels	42.5(10.4)	42.2(11.8)	45.0(10.0)	N/A	N/A	N/A	N/A

*ACS, acute coronary syndrome; STEMI, ST elevation myocardial infarction; NSTEMI, non-ST-elevation myocardial infarction; UA, unstable angina; SD, standard deviation; SBP, systolic blood pressure; DBP, diastolic blood pressure; TC, total cholesterol; TG, triglyceride; IQR, interquartile range; HDL-C, high-density lipoprotein cholesterol; LDL-C, high-density lipoprotein cholesterol; GLU, glucose levels upon admission; NLR, neutrophil-lymphocyte ratio; PLR, platelet-lymphocyte ratio; SII, immune inflammation index; ALB, serum albumin; DD, plasma D-dimer; NT-pro BNP, N-Terminal-pro-B-Type Natriuretic Peptide; cTnI, cardiac troponin I; CK, ckinase; CK-MB, ckinase-MB; LVEF, left ventricular ejection fraction; 1-VD, single-vessel disease; 2-VD, two-vessel disease; 3-VD, three-vessel disease; LAD, left anterior descending; LCX, left circumflex artery; RCA, right coronary artery; N/A, not applicable. ^a^Controls vs. STEMI, ^b^Controls vs. NSTEMI, ^c^Controls vs. UA.*

### Dynamic Changes of Biochemical Indexes in ACS Patients

The biochemical indexes in ACS patients upon admission (before PCI) and after PCI are summarized in Table 2. Most clinical biomarkers of ACS patients, except TG, HDL-C, and LDL-C, were markedly elevated within 1 day after PCI and returned to near control levels within 2 days after PCI. There was an obvious decrease in Grace scores in ACS patients within 1 week after PCI. All patients used dual antiplatelet medications after hospitalization, and more than half of them were given β-blockers (83.7%), nitrates (97.8%), and statin (97.8%) therapy.

**TABLE 2:** The dynamic changes of biochemical indexes in ACS patients before and after PCI.

**Variables**	**Before PCI (*n* = 92)**	**1 day after PCI (*n* = 53)**	**2 days after PCI (*n* = 20)**	**3 days after PCI (*n* = 22)**	**4 to 7 days after PCI (*n* = 28)**	***p*^a^**	***p*^b^**	***p*^c^**	***p*^d^**
TC, mmol/l, mean (SD)	4.49(0.97)	4.12(0.78)	3.82(0.48)	3.69(0.99)	3.79(0.74)	<0.001	0.180	0.180	0.423
TG, mmol/l, median (IQR)	1.54(1.01–2.08)	1.45(1.02–1.86)	1.20(1.10–2.47)	1.05(0.86–1.95)	1.88(1.27–4.89)	0.078	0.655	0.655	0.317
HDL-C, mmol/l, mean (IQR)	0.96(0.89–1.22)	0.97(0.85–1.16)	0.98(0.90–1.26)	0.89(0.73–1.09)	0.83(0.74–0.95)	0.593	0.910	0.500	0.500
LDL-C, mmol/l, mean (SD)	2.54(0.69)	2.49(0.70)	2.53(0.80)	2.18(0.92)	2.28(0.98)	0.109	0.689	0.500	0.500
GLU, mmol/L, median (IQR)	7.95(6.13–11.15)	6.60(5.70–8.73)	6.85(5.55–10.25)	6.90(5.75–10.30)	6.70(6.00–8.70)	0.002	0.575	0.009	0.044
NLR, median (IQR)	3.40(2.06–6.41)	4.60(2.93–6.27)	4.25(2.27–5.09)	4.44(2.32–5.83)	4.22(2.87–5.83)	0.975	0.314	0.112	0.983
PLR, median (IQR)	114.7(79.7–178.7)	127.8(99.57–175.4)	156.6(98.49–192.4)	127.5(82.53–172.8)	137.4(108.7–177.7)	0.213	0.721	0.286	0.244
SII, median (IQR)	689.8(423.4–1203.7)	880.2(671.6–1239.7)	688.8(440.1–1414.2)	941.3(526.5–1203.0)	876.7(707.7–1338.9)	0.765	0.722	0.255	0.841
ALB, g/L, median (IQR)	41.60(37.65–43.90)	38.50(36.75–41.20)	37.50(35.90–40.90)	38.60(33.48–40.30)	39.15(35.52–40.80)	0.001	0.161	0.084	0.003
DD, mg/L, median (IQR)	0.32(0.19–0.50)	0.38(0.23–1.18)	0.48(0.27–0.93)	0.26(0.18–1.04)	0.45(0.22–0.97)	0.170	0.068	0.917	0.260
NT-pro-BNP, pmol/L, median (IQR)	31.30(6.38–159.73)	87.20 (47.80–219.00)	169.40 (116.20–302.50)	130.75(39.13–498.03)	77.80(34.70–252.48)	0.011	0.114	0.239	0.012
cTnI, ng/ml, median (IQR)	0.25(0.05–5.89)	23.98(11.90–50.00)	16.86(0.57–42.88)	5.46(2.34–23.52)	5.47(1.01–9.83)	<0.001	0.161	0.314	0.196
CK, IU/L, median (IQR)	113.0(75.50–322.00)	975.00(147.00–2373.00)	262.00(84.25–1168.25)	210.00(50.00–402.00)	120.00(85.00–237.00)	0.001	0.594	0.917	0.841
CK-MB, IU/L, median (IQR)	16.00(6.00–38.00)	100.00 (21.00–211.00)	32.00(7.25–65.50)	15.00(7.50–25.00)	11.00(8.00–15.75)	0.001	0.814	0.349	0.513
Grace scores, mean (SD)	144.0(33.0)	104.6(23.8)	110.4(30.0)	108.6(27.1)	99.5(25.8)	<0.001	<0.001	<0.001	<0.001

*PCI, percutaneous coronary intervention; SD, standard deviation; IQR, interquartile range; TC, total cholesterol; TG, triglyceride; HDL-C, high-density lipoprotein cholesterol; LDL-C, high-density lipoprotein cholesterol; GLU, glucose levels upon admission; NLR, neutrophil-lymphocyte ratio; PLR, platelet-lymphocyte ratio; SII, immune inflammation index; ALB, serum albumin; DD, plasma D-dimer; NT-pro BNP, N-Terminal-pro-B-Type Natriuretic Peptide; cTnI, cardiac troponin I; CK, ckinase; CK-MB, ckinase-MB; ^a^Before PCI vs. 1 day after PCI, ^b^Before PCI vs. 2 days after PCI, ^c^Before PCI vs. 3 days after PCI, ^d^Before PCI vs. 4 to 7 days after PCI.*

### Dynamic Changes of Serum miR-186-5p Levels in ACS Patients

**FIGURE 1:**
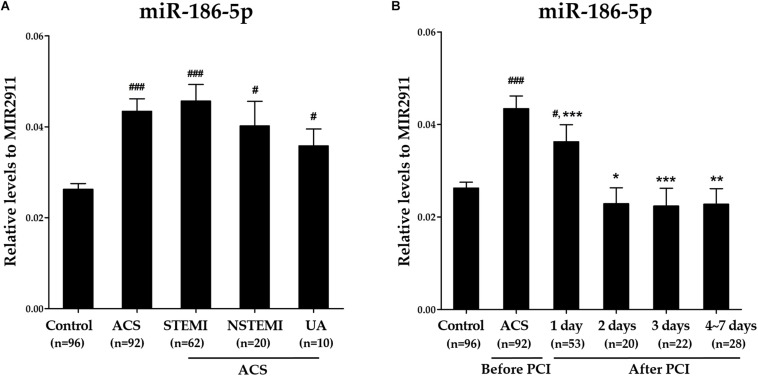
The dynamic changes of serum miR-186-5p levels in ACS patients before and after PCI. **(A)** Relative levels of serum miR-186-5p in controls and ACS patients (STEMI, NSTEMI, and UA patients). Cq values of exogenous MIR2911 showed no significant differences between the control group and each patient group. Compared with the control group, ^#^*p* < 0.05, ^###^*p* < 0.001. **(B)** Relative levels of serum miR-186-5p in ACS patients upon admission (before PCI) and within 1 week after PCI. Cq values of serum miR-186-5p were converted to relative levels normalized to spiked-in exogenous MIR2911 using the comparative Cq method (2^∧−ΔCq^). Compared with the before PCI group, ^*^p < 0.05, ^∗∗^*p* < 0.01, ^∗∗∗^*p* < 0.001.

Compared with the controls, relative levels of serum miR-186-5p were significantly increased in STEMI, NSTEMI and UA patients, while no significant differences were observed among these three subgroups (Supplementary Figure S1A and Figure 1A). However, high serum miR-186-5p levels in ACS patients upon admission (before PCI) were significantly decreased at all time points within 1 week after PCI and returned to near control levels within 1∼2 days after PCI (Figure 1B).

To further verify the above findings, we calculated the absolute concentrations of miR-186-5p by establishing calibration curves with corresponding synthetic mature miR-186-5p oligonucleotides. Consistently, absolute concentrations of serum miR-186-5p exhibited the same tendencies as normalization to the exogenous control (MIR2911), which further confirmed our results (Supplementary Figures S1B, S2).

### Levels of miR-186-5p in Serum and Myocardial Tissues of AMI Rats

**FIGURE 2:**
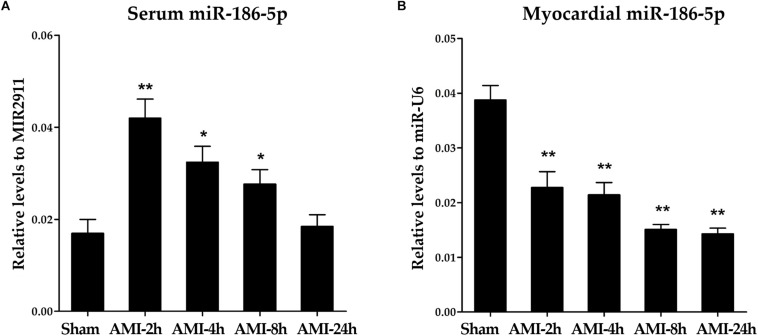
miR-186-5p levels in serum and myocardial tissues of AMI rats. **(A)** Relative levels of miR-186-5p in serum of AMI rats (*n* = 5 per group). Cq values of miR-186-5p were converted to relative levels normalized to spiked-in exogenous MIR2911 using the comparative Cq method (2^∧−ΔCq^). Cq values of the exogenous MIR2911 in serum samples showed no significant difference among five groups. **(B)** Relative levels of miR-186-5p in ischemic myocardium of AMI rats (*n* = 5/group). Cq values of miR-186-5p were converted to relative levels normalized to endogenous U6 using the comparative Cq method (2^∧−ΔCq^). Cq values of endogenous U6 in myocardial tissues showed no significant difference among five groups. Compared with the sham-operated group, ^*^p < 0.05; ^∗∗^*p* < 0.01.

Relative levels of miR-186-5p were significantly increased in serum samples from rats following LAD ligation compared those in sham-operated rats (Supplementary Figure S3A and Figure 2A). Conversely, relative levels of miR-186-5p were significantly decreased in myocardial tissues from rats following LAD ligation compared with those in sham-operated rats (Supplementary Figure S3B and Figure 2B). Strikingly, elevated miR-186-5p levels in the serum of rats rapidly reached peak values at 2 h after LAD ligation and declined at subsequent time points.

### Identification and Characterization of Serum-Derived Exosomes

To further explore the predominant form of serum miR-186-5p, we separated exosomes from serum samples of 35 controls and 35 ACS patients [including their serum samples upon admission (before PCI) and within 1 day after PCI]. The morphology type of isolated microvesicles were analyzed by NTA and TEM, and these results revealed a group of particles with a predominant size of approximately 120 nm (Supplementary Figure S4A) and several with a round, cup-shaped morphology typical of exosomes (Supplementary Figure S4B). Western blotting demonstrated that the isolated particles expressed all three characteristic exosome markers (Supplementary Figure S4C). All together, these results suggested that the main contents of the isolated microvesicles were exosome particles.

### Predominant Form of Serum miR-186-5p

**FIGURE 3:**
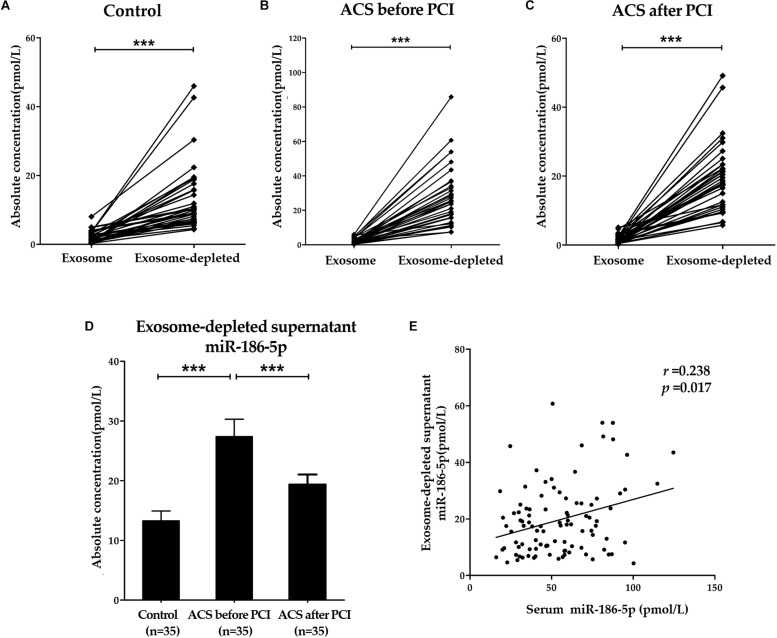
Predominant form of serum miR-186-5p. **(A–C)** Absolute concentrations of miR-186-5p in exosome fractions and exosome-depleted supernatants from serum samples of controls and ACS patients. Each point represents the mean of triplicate samples. **(D)** Comparison of absolute concentrations of miR-186-5p in exosome-depleted supernatants from serum samples of controls and ACS patients. **(E)** Correlations between relative miR-186-5p levels in total serum samples and absolute miR-186-5p concentrations in exosome-depleted supernatants (*r* = 0.238, *p* = 0.017). ^∗∗∗^*p* < 0.001.

As shown in Figures 3A–C, absolute miR-186-5p concentrations in exosome-depleted supernatants were significantly higher than those in isolated exosomes from the same serum samples of either ACS patients or controls. Absolute miR-186-5p concentrations in exosome-depleted supernatants accounted for the majority of their total concentrations in the serum samples from all studied groups. The median proportions of absolute miR-186-5p concentrations in exosome-depleted supernatants from serum samples were 85.9% (IQR, 72.8 ∼ 92.8%) in the control group, and 93.5% (IQR, 90.5 ∼ 96.8%) in the upon admission (before PCI) group, were, and 93.0% (IQR, 90.0 ∼ 96.1%) in the after PCI group.

Similarly, absolute miR-186-5p concentrations in exosome-depleted supernatants from serum samples of ACS patients upon admission (before PCI) were significantly elevated compared with those of controls, but their concentrations were also decreased after PCI (Figure 3D). However, there was no significant difference in the miR-186-5p concentrations among the three cohorts in the exosome fraction. Unsurprisingly, Spearman’s rank correlation analyses in all samples (*n* = 105) revealed that total serum miR-186-5p concentrations were positively related with miR-186-5p concentrations in exosome-depleted supernatants (*r* = 0.238, *p* = 0.017), but not in exosomes (*r* = 0.132, *p* = 0.190) (Figure 3E).

### Correlations of Serum miR-186-5p With Biochemical Parameters in ACS Patients

Spearman’s rank correlation analyses showed that serum miR-186-5p levels in ACS patients upon admission (before PCI) were positively correlated with LDL-C, NLR, DD and Gensini scores and negatively correlated with HDL-C levels. Serum miR-186-5p levels in ACS patients within 1 day after PCI were positively correlated with LDL-C, NLR, DD and Grace scores and negatively correlated HDL-C levels and LVEF after PCI. And there was no statistical correlation between serum miR-186-5p levels with LVEF and NT-pro-BNP in ACS patients at follow up (Supplementary Table S3). Then, to further analyze the relationship between serum miR-186-5p and patient disease progression, we calculated the serum miR-186-5p and clinical marker differences between after PCI and before PCI; Δserum miR-186-5p, ΔcTNI, ΔCK-MB, and ΔNT-pro-BNP were calculated by subtracting the biomarker levels after PCI from those before PCI. Surprisingly, we found that Δserum miR-186-5p had strong positive correlations with ΔcTNI and Gensini scores (Table 3).

**TABLE 3:** Correlations of serum miR-186-5p and biochemical parameters in ACS patients.

**Variables**	**Serum miR-186-5p**		**Variables**	**Serum miR-186-5p**		**Variables (Δ)**	**ΔSerum miR-186-5p**	
**(before PCI)**	**(before PCI)**		**(after PCI)**	**(after PCI)**				
HDL-C	*r* = −0.443	*p* = 0.034	HDL-C	*r* = −0.393	*p* = 0.043	ΔHDL-C	*r* = −0.055	*p* = 0.734
LDL-C	*r* = 0.522	*p* = 0.009	LDL-C	*r* = 0.460	*p* = 0.016	ΔLDL-C	*r* = 0.550	*p* = 0.667
NLR	*r* = 0.226	*p* = 0.040	NLR	*r* = 0.367	*p* = 0.035	ΔNLR	*r* = 0.313	*p* = 0.092
PLR	*r* = −0.124	*p* = 0.245	PLR	*r* = 0.255	*p* = 0.128	ΔPLR	*r* = 0.056	*p* = 0.743
SII	*r* = 0.047	*p* = 0.663	SII	*r* = 0.215	*p* = 0.221	ΔSII	*r* = 0.172	*p* = 0.322
DD	*r* = 0.261	*p* = 0.030	DD	*r* = 0.537	*p* = 0.048	ΔDD	*r* = −0.552	*p* = 0.063
cTNI	*r* = 0.054	*p* = 0.644	cTNI	*r* = 0.017	*p* = 0.927	ΔcTNI	*r* = 0.435	*p* = 0.018
CK-MB	*r* = 0.062	*p* = 0.575	CK-MB	*r* = −0.258	*p* = 0.135	ΔCK-MB	*r* = 0.116	*p* = 0.548
Grace scores	*r* = 0.115	*p* = 0.274	Grace scores	*r* = 0.340	*p* = 0.016	ΔGrace scores	*r* = 0.058	*p* = 0.687
Gensini scores	*r* = 0.208	*p* = 0.046	N/A	N/A	N/A	Gensini scores	*r* = −0.346	*p* = 0.011
N/A	N/A	N/A	LVEF	*r* = −0.358	*p* = 0.044	LVEF	*r* = 0.247	*p* = 0.173

*PCI, percutaneous coronary intervention; HDL-C, high-density lipoprotein cholesterol; LDL-C, high-density lipoprotein cholesterol; NLR, neutrophil-lymphocyte ratio; PLR, platelet-lymphocyte ratio; SII, immune inflammation index; DD, plasma D-dimer; cTnI, cardiac troponin I; CK, ckinase; CK-MB, ckinase -MB; LVEF, left ventricular ejection fraction after PCI; N/A, not applicable.*

### Predictive Values of Serum miR-186-5p in ACS Patients

**FIGURE 4:**
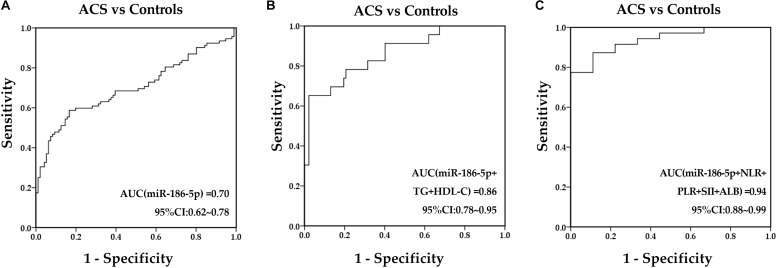
Predictive values of serum miR-186-5p and clinical markers to predict ACS presence. **(A)** ROC curves for serum miR-186-5p. **(B)** ROC curves for the combination of miR-186-5p with TG and HDL-C. **(C)** ROC curves for the combination of miR-186-5p with NLR, PLR, SII, and ALB.

Receiver-operating characteristic analysis was performed to determine the discriminative ability of serum miR-186-5p and other indexes before PCI for identifying ACS from controls. As a result, serum miR-186-5p, GLU, TG, HDL-C, NLR, PLR, SII, and ALB were revealed to have predictive value for ACS and normal controls. The AUC of serum miR-186-5p was 0.70 (95% CI, 0.62∼0.78, *p* < 0.001) (Figure 4A), and the AUCs of NLR, PLR, SII, and ALB before PCI were 0.80 (95% CI, 0.73∼0.87, *p* < 0.001), 0.59 (95% CI, 0.51∼0.68, *p* = 0.039), 0.78 (95% CI, 0.71∼0.85, *p* < 0.001) and 0.81 (95% CI, 0.71∼0.91, *p* = 0.002), respectively. Next, we calculated the predicted probability of serum miR-186-5p and these indexes using the binary logistic regression analyses. An ROC curve of their combination was generated based on these probabilities. The AUC was improved after combining miR-186-5p with lipid parameters (TG and LDL-C), inflammation indexes (NLR, PLR, SII and ALB) (Figures 4B,C) and GLU (Supplementary Figure S5). Additionally, the binary logistic regression analyses were performed to assess the predictive values of serum miR-186-5p for the presence of ACS. The ACS or control group was treated as a dependent two-category variable, and the reference category was the control group. The univariate analysis indicated that high levels of serum miR-186-5p may be closely associated with ACS presence, and its odds ratio (OR) was 1.04 (95% CI, 1.03∼1.07, *p* < 0.001). The univariate logistic analysis also showed that NLR, PLR, SII, and ALB might be independent predictors for ACS presence (all *p* < 0.05). After adjusting for age, gender, blood pressure, coexisting conditions (hypertension, diabetes mellitus, dyslipidemia, tobacco use), lipid profiles (TC, TG, HDL-C, and LDL-C), multivariate analysis revealed that serum miR-186-5p may be considered an independent predictor of ACS presence (ORs = 1.11, 95% CI, 1.00∼1.23, *p* = 0.043), and inflammatory indexes (NLR, PLR SII and ALB) showed no predictive values (all *p* > 0.05).

### Prognostic Values of Serum miR-186-5p in ACS Patients

**FIGURE 5:**
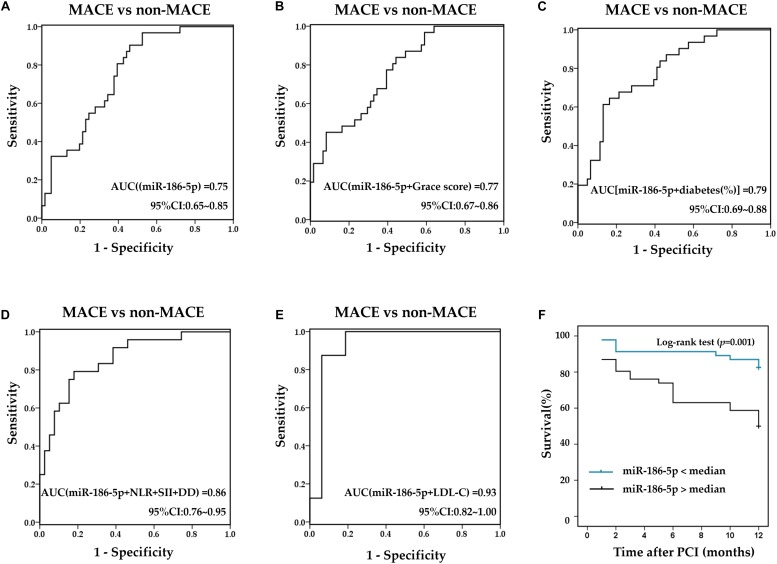
Prognostic values of serum miR-186-5p and clinical markers. **(A)** ROC curves for serum miR-186-5p. **(B–E)** ROC curves for the combination of miR-186-5p with clinical markers **(B)** Combination of miR-186-5p with Grace scores, **(C)** combination miR-186-5p with proportions of diabetes, **(D)** combination miR-186-5p with NLR, SII and DD, **(E)** combination of miR-186-5p with LDL-C to predict the occurrence of one-year MACE. **(F)** Kaplan Meier survival curves stratified by miR-186-5p levels in ACS patients undergoing PCI.

Within one -year of follow-up, the incidence of MACE events in ACS patients was 33.7% (a total of 31 cases), including 8 cases of target vessel revascularization, 19 cases of non-fatal MI, 1 case of cardiac death and 3 cases of stroke, and the majority of MACE occurred within the first 3 months after PCI [15 cases (48.4%)]. ACS patients with Gensini scores above the median showed a significantly higher incidence of MACE (χ^2^ = 3.941, *p* = 0.047). The percentage of diabetes in the MACE group (61.3%) was significantly higher than that in the non-MACE group (34.4%) (χ^2^ = 6.036, *p* = 0.014). Grace scores after PCI in ACS patients with MACE were significantly higher than those in ACS patients without MACE (*t* = −2.815, *p* = 0.006). In addition, relative serum miR-186-5p levels upon admission (before PCI) in ACS patients with MACE [0.053 (0.038∼0.076)] were significantly higher than that in ACS patients without MACE [0.028 (0.017∼0.050)] (*Z* = −3.903, *p* < 0.001). Moreover, ACS patients with MACE exhibited markedly higher NLR, SII and DD levels before PCI than patients without MACE (*Z* = −2.865, *p* = 0.004; *Z* = −2.719, *p* = 0.007; *Z* = −2.442, *p* = 0.015). Furthermore, ROC curves were calculated to compare the differential values of miR-186-5p and other clinical biomarkers for the occurrence of MACE. The AUC of serum miR-186-5p, Grace scores, LDL-C, NLR, SII before PCI and proportions of diabetes were 0.75 (95% CI, 0.65∼0.85, *p* < 0.001) (Figure 5A), 0.66 (95% CI, 0.55∼0.78, *p* = 0.010), 0.86 (95% CI, 0.71∼1.00, *p* = 0.004), 0.74 (95% CI, 0.61∼0.86, *p* = 0.001), 0.68 (95% CI, 0.56∼0.80, *p* = 0.001), and 0.63 (95% CI, 0.51∼0.76, *p* = 0.036), respectively. Additionally, the prognostic prediction efficiency was further improved after combining miR-186-5p with the above markers (Figures 5B–E). However, the AUC of serum NT-pro-BNP [0.51 (95% CI, 0.35∼0.67, *p* = 0.080)], cTNI [0.47 (95% CI, 0.31∼0.62, *p* = 0.656)], and CK-MB [0.44 (95% CI, 0.31∼0.56, *p* = 0.366)] before PCI as well as Gensini scores before PCI [0.61 (95% CI, 0.49∼0.72, *p* = 0.098) showed no statistical significance. After dividing patients into 2 groups according to the median serum miR-186-5p concentration upon admission, miR-186-5p content above median were associated with a significantly higher incidence of MACE as well as non-fatal-MI (χ^2^ = 6.343, *p* = 0.012; χ^2^ = 10.87, *p* = 0.001). Kaplan-Meier survival analysis showed that the event-free survival rate of the high miR-186-5p group was lower than that of the low miR-186-5p group during the follow-up (log-rank = 11.14, *p* = 0.001) (Figure 5F).

Furthermore, univariate and multivariate Cox regression analyses were conducted to evaluate the prognostic values of serum miR-186-5p for the occurrence of one-year MACE in ACS patients after PCI. The univariate analysis indicated a strong correlation between increased serum miR-186-5p levels and the occurrence of one-year MACE in ACS patients after PCI (HR = 1.03, 95% CI, 1.01∼1.04, *p* = 0.029). And NLR, SII, and DD before PCI were closely related to the occurrence of MACE (HR = 1.14, 95% CI, 1.03∼1.25 *p* = 0.012; HR = 1.01, 95% CI, 1.00∼1.02 *p* = 0.012; HR = 1.60, 95% CI, 1.04∼2.47, *p* = 0.033, respectively). After adjusting for age, sex, ACS diagnosis, coexisting conditions (hypertension, diabetes mellitus, dyslipidemia, tobacco use) and clinical markers (TC, TG, NT-pro-BNP, cTNI, and CK-MB), the multivariate analysis revealed that NLR, SII, and DD showed no prognostic values (all *p* > 0.05) and serum miR-186-5p may be considered an independent prognostic factor for the occurrence of one-year MACE in ACS patients after PCI (HR = 1.13, 95% CI, 1.03∼1.24, *p* = 0.008).

### Analysis of miR-186-5p Target Genes

**FIGURE 6:**
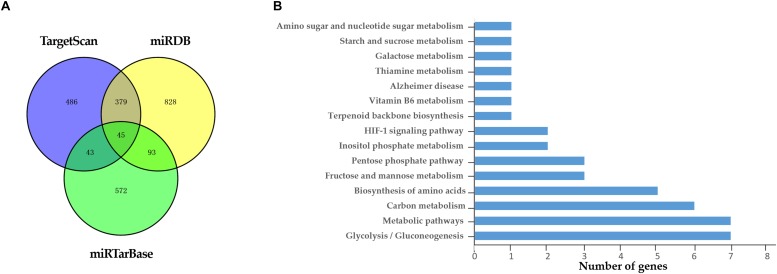
Target genes of miR-186-5p. **(A)** Venn diagram: the number of candidate common target genes determined by three (TargetScan, miRTarBase, and miRDB) bioinformatics analyses. **(B)** KEGG Enrichment Pathways.

A total of 45 target genes were predicted by the combination of three different bioinformatics approaches (Figure 6A). The result of KEGG enrichment pathway analysis was enumerated in Figure 6B. At least 15 target genes of miR-186-5p were involved in carbohydrate metabolism, such as the glycolysis/gluconeogenesis pathway, pentose phosphate pathway and starch and sucrose metabolism pathway. The other genes were involved in HIF-1 signaling pathway and other metabolic pathways. Therefore, miR-186-5p might participate in the development of ACS via affecting glucose metabolism, the hypoxia response and so on.

## Discussion

Circulating miRNAs have been proposed as potential biomarkers for the early prediction for cardiovascular events. Emerging evidence indicates that specific miRNAs exhibit altered expression signatures in ACS patients before and after PCI treatment (Chen et al., 2015; Yang et al., 2016; Zhu et al., 2016; Zhang et al., 2018), suggesting that distinctive miRNA profiles may also conveniently and effectively offer information reflecting the disease progression of ACS patients after reperfusion therapy. Serum/plasma miR-186-5p levels have been reported to notably increase in AMI and UA patients (Wu et al., 2014; Zeller et al., 2014; Liu X. et al., 2016). However, there still has been no research on the dynamic changes in circulating miR-186-5p expression pattern in ACS patients before and after PCI. In the present study, we found that serum miR-186-5p levels were significantly elevated in ACS patients upon admission compared with those in controls. This result was consistent with the findings from previous studies (Wu et al., 2014; Zeller et al., 2014; Liu X. et al., 2016). Furthermore, we observed that serum miR-186-5p levels in ACS patients upon admission were positively correlated with initial Gensini scores. This has proven that specific miRNAs may participate in plaques rupture and endothelial erosion, leading to an abrupt transition from asymptomatic atherosclerosis or stable CAD to ACS (Libby, 2013; Jin et al., 2018). Recently, Yao et al. (2016) revealed that miR-186-5p promoted the accumulation of atherogenic lipids and increased the secretion of proinflammatory cytokines in macrophages, which may accelerate the progression of atherosclerotic lesions and eventually cause the onset of ACS. Thus, we supposed that elevated serum levels of miR-186-5p in ACS patients might serve as potential indicators of aggravated plaque vulnerability and consequently be associated with the severity of advanced lesions. Strikingly, we also provided the first evidence that initially high miR-186-5p levels in ACS patients were gradually decreased within 1 week after PCI and returned to near control levels within 1∼2 days after PCI. Furthermore, we uncovered that serum miR-186-5p levels in ACS patients after PCI were positively correlated with Grace scores and negatively correlated with initial LVEF. It is well known that timely coronary artery recanalization and myocardial reperfusion could effectively limit the extent of necrosis and infarct size. Altered serum miR-186-5p levels in ACS patients before and after PCI suggested that successful medical intervention could promptly rescue myocardial tissues and preserve cardiac function, ultimately improving clinical outcomes. Therefore, serum miR-186-5p may act as a reliable biomarker for monitoring the clinical condition and assessing the prognosis of ACS patients.

Currently, most existing studies have identified that miRNAs encapsulated within exosomes or microvesicles may contribute to cardiac pathophysiology via targeting various cell types, such as endothelial cells and myocardial cells (Halkein et al., 2013; Barile et al., 2017). Nevertheless, extracellular circulating miRNAs also exist with free-floating status and a protected by RNA-binding proteins, such as nucleophosmin 1, HDL and Ago2 (Xu et al., 2013). In view of the kinetic signatures of serum miR-186-5p in ACS patients before and after PCI, we further analyzed the potential source and predominant form of serum miR-186-5p. We also observed that serum miR-186-5p mainly existed in an exosome-free form rather than membrane-bound exosomes in both ACS patients and controls. Moreover, the changes in absolute miR-186-5p concentrations in exosome-depleted supernatants from serum samples of ACS patients on admission exhibited similar trends as those observed for total miR-186-5p concentrations in corresponding serum samples. It seems reasonable to hypothesize that miR-186-5p might be passively released from ischemic myocardium rather than actively secreted via exosomes. Most of the published studies have proven that cardiac-derived miRNAs (e.g., miR-1/133a-b/208a/499) are pivotal biomarkers for AMI (Wang et al., 2010). In the present study, we observed no signature relationship between miR-186-5p and direct concentrations of cTnI, CK-MB while the differences in serum miR-186-5p levels showed a positive correlation in the changes in cTNI after PCI treatment, which may result from the different kinetics among these factors. Wang et al. (2010), Liu X. et al. (2016) found that miR-186-5p reached peak expression at an earlier time (4 h after the onset of chest pain) than cTnI expression (8 h after chest pain). In our study, logistic regression analyses analysis revealed that serum miR-186-5p may be considered an independent risk factor for ACS presence, and the current study indicated the potential utility of serum miR-186-5p as an ischemia/reperfusion monitoring biomarker of ACS patients via PCI.

Coronary stenosis and/or complete obstruction of single vessels or multiple vessels could trigger ACS and affect the prognosis of patients (Marfella et al., 2013b; Marfella et al., 2018a, 2018b). We found that ACS patients with more severe stenosis of the coronary arteries showed upregulated serum miR-186-5p levels as well as a higher incidence of MACE after PCI. ACS is mainly caused by atherosclerosis, which is considered a chronic inflammatory response to vaso-active stimuli. Inflammatory disorders in coronary thrombi might produce high thrombus burdens, leading to reduced coronary flow reserve and undesirable outcomes in ACS patients (Sardu et al., 2018, 2019). Recently, several new white blood -cell-based inflammatory indexes, such as NLR, PLR and SII, have emerged as indicators for patients with cardiac disease and showed good associations with prognosis for STEMI (Sawant et al., 2014; Fest et al., 2018). In the present study, ROC analyses indicated that NLR and SII could serve as an independent predictive and prognostic factors for ACS, which was consistent with previous studies. Moreover, serum miR-186-5p correlated strongly with elevated LDL-C and NLR, and the predictive power of miR-186-5p was significantly improved for the presense of ACS and one-year MACE occurrence when combining miR-186-5p with NLR, PLR, SII, and LDL-C. Excessive vascular inflammation, endothelial dysfunction and thrombogenic tendency can also be induced by insulin resistance and hyperglycemia (Low Wang et al., 2016; Katakami, 2018). Diabetic patients might have a higher prevalence of multivessel disease, and glucose-lowering medication decreased the mortality and MI rate in CAD patients with type 2 diabetes (Gyldenkerne et al., 2019; Sasso et al., 2019). Consistently, ACS patients with diabetes showed increased NLR and SII levels as well as higher proportions of 3-VD and MACE, and ACS patients with diabetes and 3-VD showed obviously higher serum miR-186-5p levels than those in other patients. Those results indicated that elevated miR-186-5p levels might affect the presence and prognosis of ACS by regulating inflammatory status and dysglycemia of patients, which was further confirmed by KEGG analysis of miR-186-5p target genes. Glycolysis/gluconeogenesis pathway was enriched for the largest number of target genes in the KEGG pathway analysis, and it has been reported that downregulation of miR-186-5p ameliorates high glucose-induced apoptosis in cardiomyocytes (Jiang et al., 2018). The HIF-1 signaling pathway plays a vital role in cellular responses in a low-oxygen environment and affects the pathogenesis of atherosclerosis via various molecular and cellular events (Jain et al., 2018). Study by Dang et al. (2017) demonstrated that miR-186 could restrain vascular endothelial cell proliferation and enhance apoptosis by targeting HIF-1α. Additionally, miR-186 could inhibit steps in cellular glycolysis, such as glucose intake, ATP and NADH production via HIF-1α regulation (Liu L. et al., 2016). Results from our study suggested that miR-186-5p might participate in the progression and prognosis of ACS by affecting glucose metabolism and hypoxia response, although the exact associated mechanisms still need to confirmed.

Percutaneous coronary intervention, the most widely used approach for myocardial revascularization of ACS, greatly reduced the risk of restenosis. However, stents can also cause plaque rupture and increase subsequent stent thrombosis due to vascular injury (Li et al., 2017). A large AtheroGene study showed that eight miRNAs, including miR-186, were able to reliably predict cardiovascular death in CAD patients during 4 years of follow-up, and the predictive power was larger in the ACS group than in the total CAD cohort (Karakas et al., 2017). A few circulating miRNAs, such as miR-133a and miR-126, have been validated to be closely associated with a future occurrence rate of MACE after PCI treatment in AMI or CAD patients (Yu et al., 2013; De Rosa et al., 2017). miRNAs might be associated with cardiac adaptive processes in faling heart patients and myocardial cell death was considered a major event in the progression and clinical prognosis of cardiovascular diseases (Marfella et al., 2013a; Sardu et al., 2014). Animal studies from Xu et al. (2017), Jiang et al. (2018) proved that miR-186-5p could protect or aggravate apoptosis in rat primary cardiomyocytes. It also has been reported that miR-186-5p is an adverse factor in ischemic stroke for inducing the apoptosis of neurons (Wang et al., 2018). In the present study, we observed that serum miR-186-5p was negatively related to LVEF, which hinted that miR-186-5p might influence the pump function of ACS patients, but more information during follow- up should be collected to explore the predictive values of miR-186-5p with cardiac function. The Grace score is a validated method to evaluate the clinical prognosis of ACS patients; however, there has been a limited clinical adoption of the Grace score due to increased data demands and the complicated calculation process (Corcoran et al., 2015). We found that serum miR-186-5p associated well with Grace scores in ACS patients after PCI, and circulating miR-186-5p was higher in subjects with an event compared with event-free subjects during one-year follow-up. The results from ROC curves showed that serum miR-186-5p had favorable predictive ability for the occurrence of MACE, and after combining with Grace scores, the prognostic prediction efficiency may be further improved. Cox regression analyses further suggested that the elevated levels of miR-186-5p may be involved in the poor prognosis for ACS patients after PCI. Those data indicate that miR-186-5p may play a more complex role in myocardial necrosis and measurement of miR-186-5p may represent a new candidate to affect poststenting pathological processes and short-term or long-term clinical outcomes of ACS patients.

Nevertheless, several limitations of our study should be acknowledged. First, this was a study involving a relatively small sample size and we conducted the follow-up of the participants for only 1 year; larger study groups and longer-term follow-up are necessary to confirm the clinical value of miR-186-5p in ACS. Second, we only examined whether miR-186-5p predominantly existed in exosome-depleted supernatant, and more experiments still are needed to examine whether circulating miR-186-5p binding with specific serum protein such as HDL and Ago2 and the clinical values of miR-186-5p concentrations in exosomes or exosome-depleted supernatants should be further explored as monitoring and prognostic factors. In addition, we observed altered miR-186-5p levels in AMI rat myocardial tissues, but the role and mechanisms in clinical prognosis in animals are still obscure. In our future study, we will conduct gain/loss of-function assays in certain cell types and overexpress/ inhibit miR-186-5p in AMI rats to substantiate the underlying mechanism and clinical function of miR-186-5p participation in the physiological and pathological processes of myocardial ischemia both *in vitro* and *in vivo.*

## Conclusion

In summary, the present study for the first time investigated the dynamic expressions of circulating miR-186-5p in ACS patients after revascularization via PCI. Our findings showed that serum miR-186-5p was predominantly present in exosome-free supernatant. The results from one-year follow-up indicated that circulating miR-186-5p may be a candidate for monitoring the clinical condition and assessing the prognosis of ACS patients. Further studies are required to extend follow-up and clarify the underlying molecular mechanism of miR-186-5p participation in the physiological and pathological processes of myocardial ischemia.

## Data Availability

All datasets generated for this study are included in the manuscript and/or the Supplementary Files.

## Ethics Statement

From January 2017 to September 2017, 92 consecutive eligible patients with ACS and 96 control subjects were enrolled. All subjects gave written informed consent in accordance with the Declaration of Helsinki. This study was approved by the Research Ethics Committee of Jinling Hospital (2015NZGKJ-018) and was performed in accordance with the Declaration of Helsinki of 1975, as revised in 2013. Male Sprague-Dawley (SD) rats (8∼10 weeks old) were obtained from the Model Animal Research Center of the Jinling Hospital (MARC, Nanjing, China), which were housed in the professional facilities of experimental animal resource. This study was carried out in accordance with the guidelines of the Institutional Animal Care and Use Committee of the Jinling Hospital. The protocol was approved by the Institutional Animal Care and Use Committee of the Jinling Hospital.

## Author Contributions

JuW, JiW, and ZL participated and conceived the study design. ZL, WW, and XC collected the data. ZL, WW, and JiW performed the experiments and analyzed the data. XC, JY, and CW interpreted and discussed the data. ZL and JiW wrote the manuscript. JiW, CW, JS, and JuW refined the final draft and revised the manuscript. All authors reviewed the final version of the manuscript.

## Conflict of Interest Statement

The authors declare that the research was conducted in the absence of any commercial or financial relationships that could be construed as a potential conflict of interest.

## Footnotes

1 http://www.gracescore.org

2 http://david.abcc.ncifcrf.gov
