# Supplementary material for: Association of Serum miR-186-5p With the Prognosis of Acute Coronary Syndrome Patients After Percutaneous Coronary Intervention
Source: Front Physiol. 2019 Jun 5;10:686. doi: 10.3389/fphys.2019.00686 (PMC6560170; doi:10.3389/fphys.2019.00686)
Supplement: Supplementary file 1 [file Data_Sheet_1.zip › Supplementary material/supplementary figures/Figure-S4-original picture/Figure-S4a--NTA of exsomes.pdf]

Electrophoresis & Brownian Motion
Video Analysis
Laser Scattering Microscopy

www.particle-metrix.de

Operator (Report): lenovo
Video Operator: lenovo

Sample Parameters
Sample Name: No5-LZL
Comment: Sample Remarks0: Sens:70,Shutter:70
Sample Remarks1:
Sample Remarks2:
Electrolyte: PBS
Temperature: 24.96 °C sensed
pH 7.4 entered
Conductivity: 15000.00 µS/cm sensed

Result (sizes in nm)

	Number	Concentration	Volume
Median (X50)	125.3	125.3	186.0
Span	52.6	52.6	108.3

Concentration: 6.2E+7 Particles / mL
Dilution Factor: 120
Original Concentration: 7.4E+9 Particles / mL

Quality
Average Counted Particles per Frame: 137
Number of Traced Particles: 2458

Measurement Parameters
Cell S/N: ZNTA

Measurement Mode: Size Distribution 5 Cycles
11 Positions

Analysis Parameters
Max Area: 1000, Min Area: 5, Min Brightness: 20

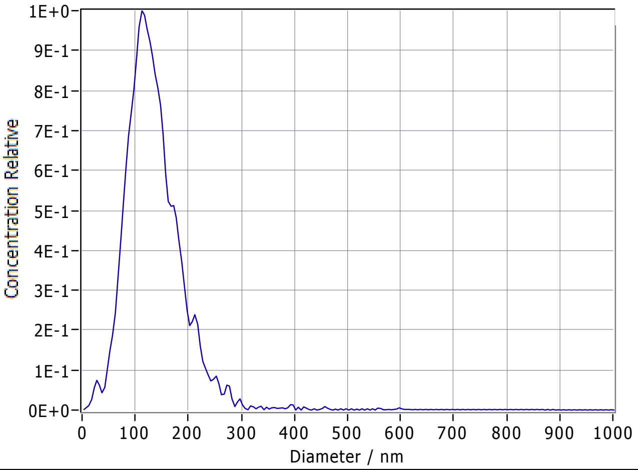

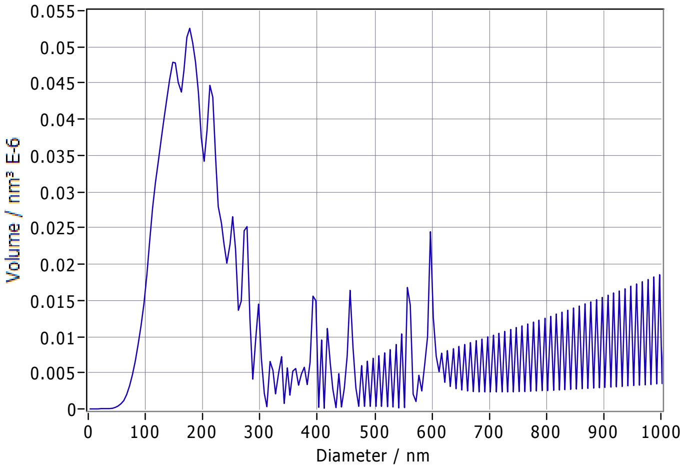

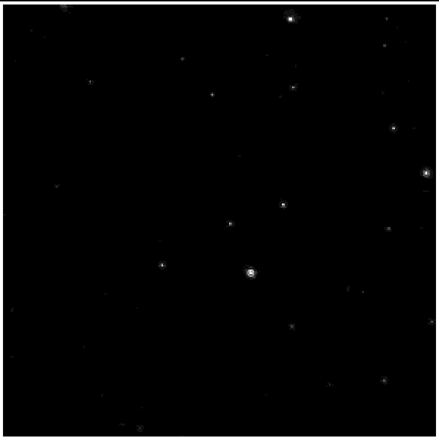

Peak Analysis (Concentration)

Diameter / nm	Particles/mL	FWHM / nm	Percentage
116.1	2.9E+6	97.9	97.8
393.0	3.9E+4	11.6	0.3
337.1	2.8E+4	30.0	0.2
457.3	2.5E+4	9.6	0.1
417.7	2.3E+4	2.1	0.1

X Values

	Number	Concentration	Volume
X10	75.4	75.4	117.5
X50	125.3	125.3	186.0
X90	196.8	196.8	365.7
Span	1.0	1.0	1.3
Mean	135.0	135.0	216.4
StdDev	52.6	52.6	108.3

Comment

(Signature)

Analyzed Video: E:\Results-17-315\NanJing Military Hospital-180604\20180604_0006_No5-LZL_size.avi

ZetaVIEW S/N 17-315, Software ZetaView 8.04.02 SP2, Camera 0.743 µm/px

Experiment: 2018-06-04 15:34, Report: 2018-06-04 15:39
