# Supplementary material for: Association of Serum miR-186-5p With the Prognosis of Acute Coronary Syndrome Patients After Percutaneous Coronary Intervention
Source: Front Physiol. 2019 Jun 5;10:686. doi: 10.3389/fphys.2019.00686 (PMC6560170; doi:10.3389/fphys.2019.00686)
Supplement: Supplementary file 1 [file Data_Sheet_1.zip › Supplementary material/Supplementary_fphys.docx]

Supplementary Material


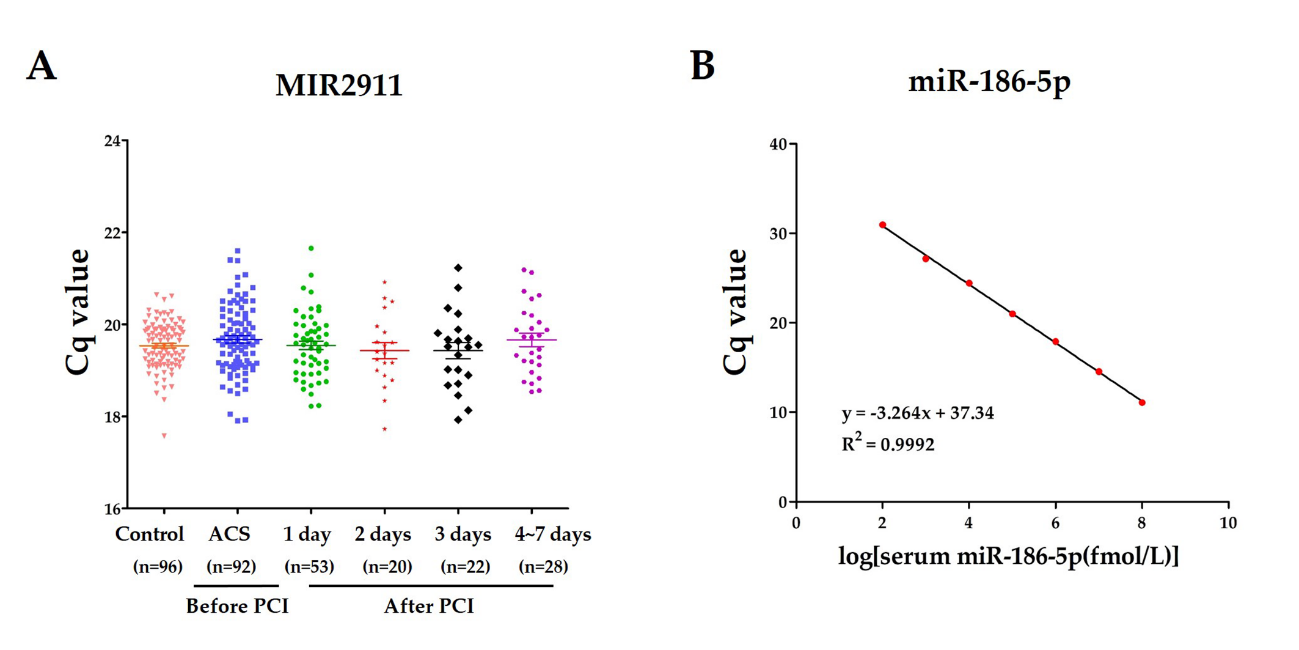


**Supplementary Figure S1.** Cq values of MIR2911 from each ACS cohort and standard curve of miR-186-5p. **(A)** Synthetic MIR 2911 was applied as a spike-in control, and we observed no significant difference among six groups. Each Cq value represents the mean of triplicate samples, and differences among those cohorts were analyzed by one-way ANOVA, *p* = 0.110. **(B)** Ten-fold serial dilution of synthetic mature miR-186-5p oligonucleotide from 10^2^ fmol/L to 10^8^ fmol/L was used to generate the standard curves. The resulting Cq values were plotted versus the log10 of the amount of each calibrators. Each point represents the mean of three independent experiments.


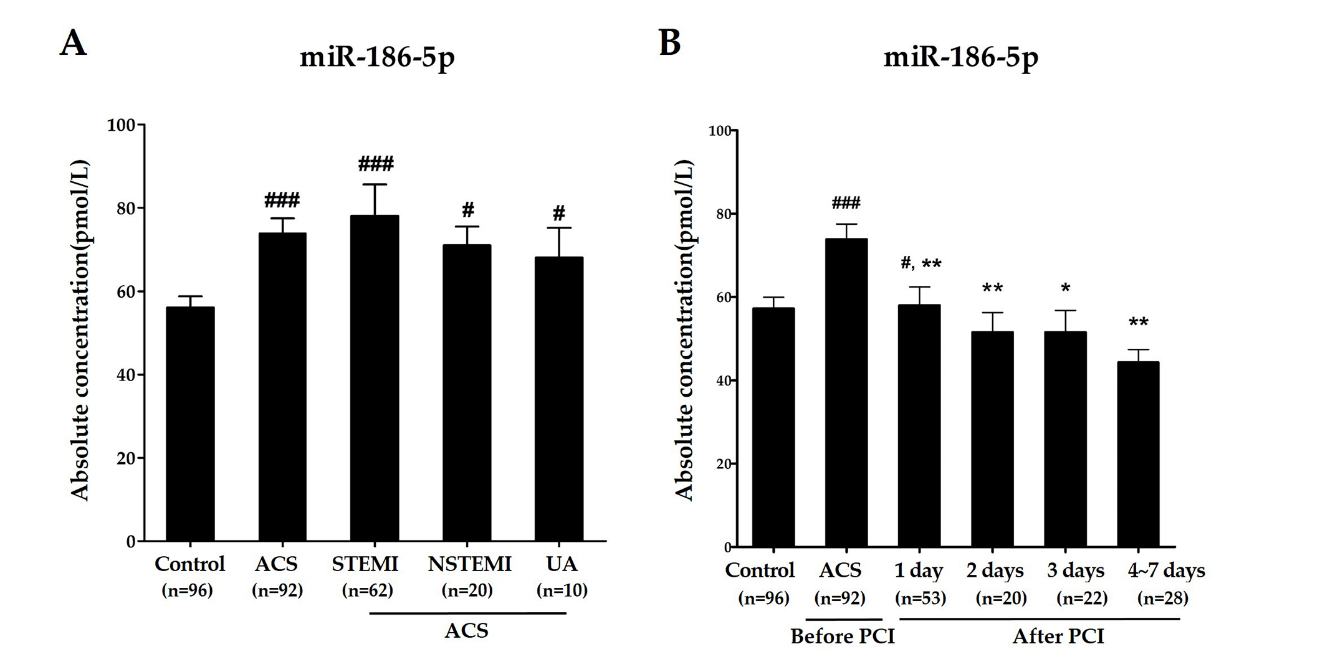


**Supplementary Figure S2.** Absolute concentrations of serum miR-186-5p in in ACS patients before and after PCI. **(A)** Absolute concentrations of serum miR-186-5p in control individuals and ACS patients (STEMI, NSTEMI and UA patients). Compared with the control group, #*p* < 0.05, ###*p* < 0.001. **(B)** Absolute concentrations of serum miR-186-5p in ACS patients on admission (before PCI) and within one week after PCI. Compared with the before PCI group, **p* < 0.05, ***p* < 0.01.


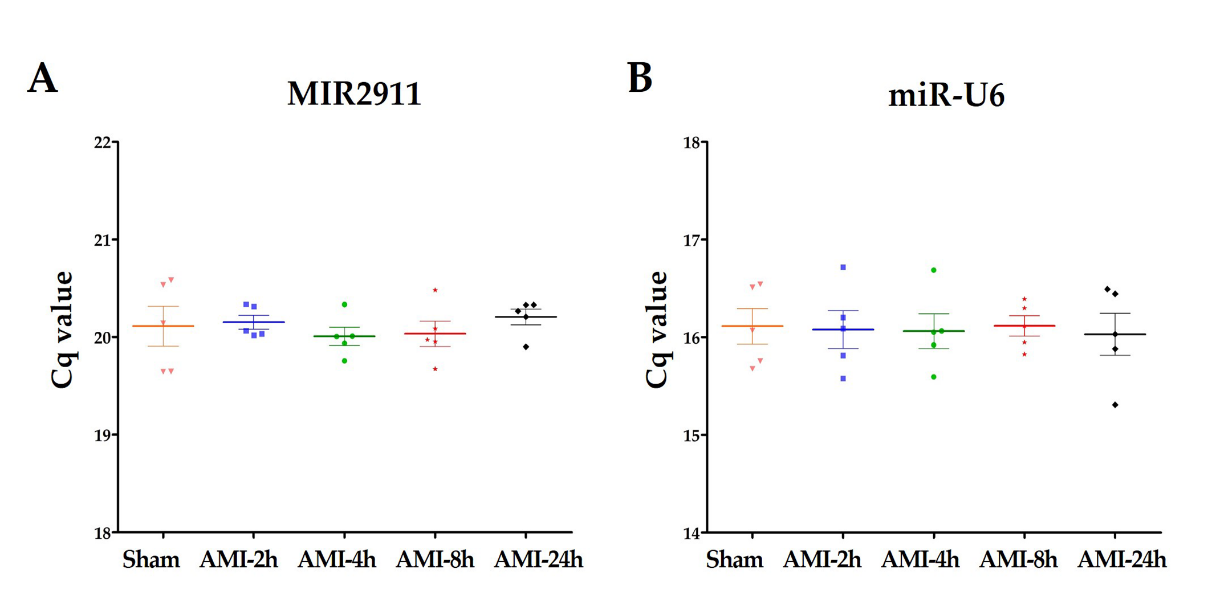


**Supplementary Figure S3.** Cq values of MIR 2911 in serum samples and miR-U6 in myocardial tissues of rats. **(A)** Cq values of the exogenous MIR2911 in serum samples had no significant difference among five groups. Each Cq value represents the mean of triplicate samples, and differences among those cohorts were analyzed by one-way ANOVA, *p* = 0.786. **(B)** Cq values of the endogenous U6 in myocardial tissues showed no significant difference among five groups. Each Cq value represents the mean of triplicate samples, and differences among those cohorts were analyzed by one-way ANOVA, *p* = 0.997.


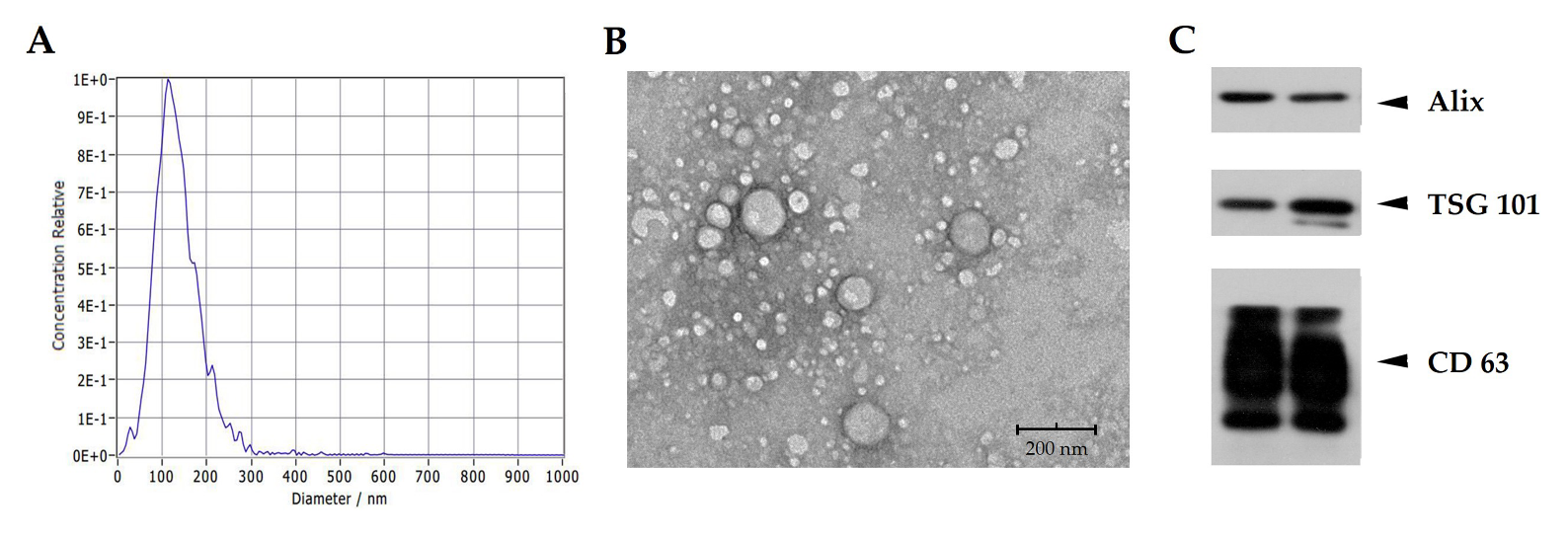


**Supplementary Figure S4.** Characterization of exosomes isolated from ACS patient serum. **(A)** Size distribution of serum exosomes as analysed by Nanoparticle Tracking Analysis. **(B)** Transmission electron micrograph of serum exosomes (scale bar: 200nm). **(C)** Western blotting for exosome-associated proteins CD63(20-70 KD), TSG 101(46 KD) and Alix (96 KD) in patient samples.


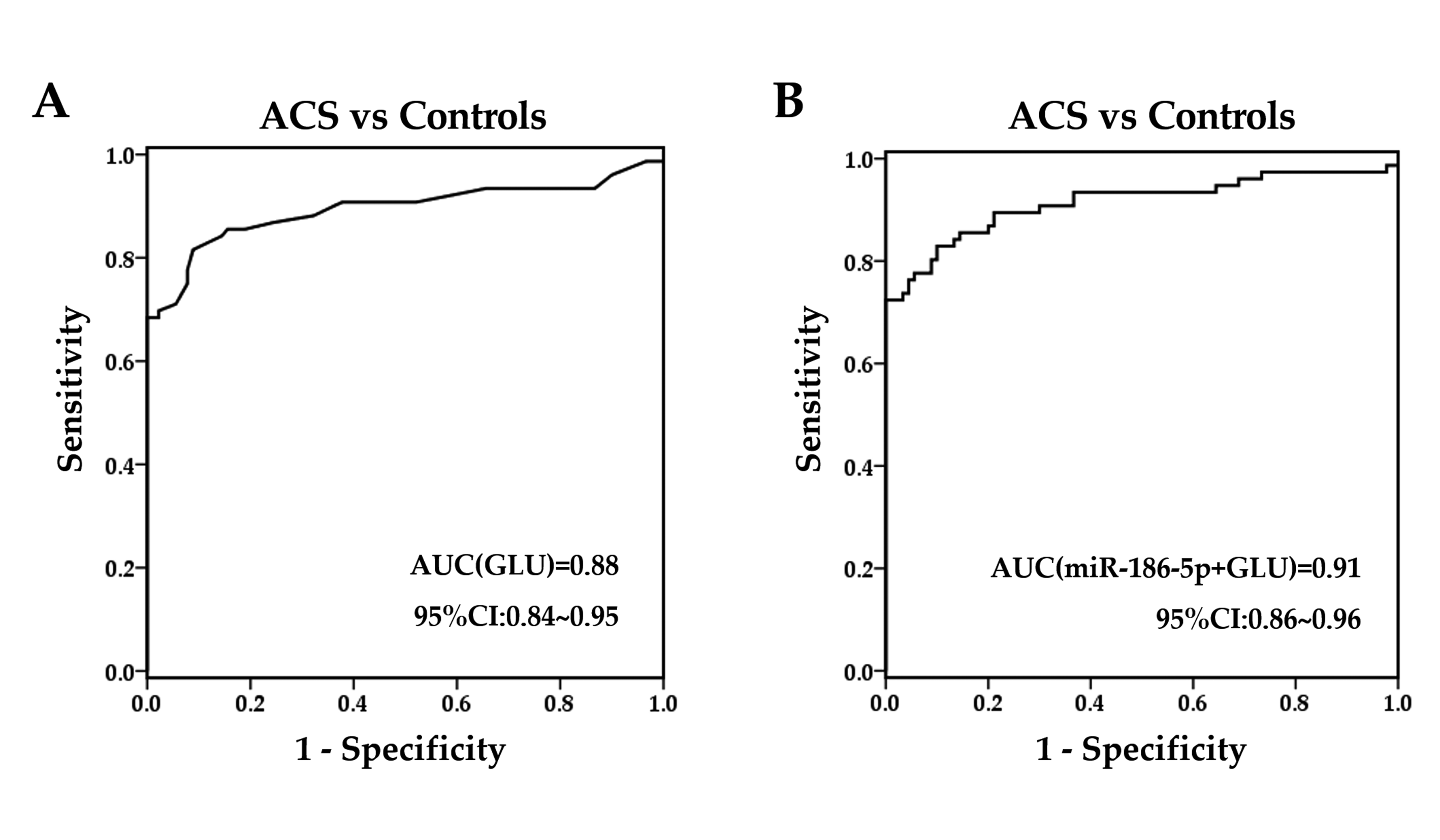


**Supplementary Figure S5.** ROC curves for GLU to predict the presence of ACS. **(A)** ROC curves for GLU (glucose levels upon admission). **(B)** ROC curves for the combination of miR-186-5p with GLU.

**Table S1**. Distribution of segments of abnormal wall motion

| **Abnormal wall motion** | **Name of abnormal segments** | | | |
| --- | --- | --- | --- | --- |
|  | **1-4** | **5-8** | **9-12** | **13-17** |
| totall abnormal segments (n) | 12 | 13 | 20 | 30 |
| hypokinesis (n) | 10 | 11 | 19 | 28 |
| akinesis (n) | 2 | 2 | 1 | 2 |
| dyskinesis (n) | 0 | 0 | 0 | 0 |

**Table S2.** Angiographic data of ACS patients before PCI and at follow up

| **Variables** | **Before PCI (n=92)** | | |  | **One-year follow up(n=22)** | | |
| --- | --- | --- | --- | --- | --- | --- | --- |
|  | **STEMI (n=62)** | **NSTEMI (n=20)** | **UA  (n=10)** |  | **STEMI (n=11)** | **NSTEMI (n=7)** | **UA  (n=4)** |
| Number of affected vessel, no (%) | |  |  |  |  |  |  |
| 1-VD | 14(22.6) | 2(10.0) | 2(20.0) |  | 2(18.18) | 0(0) | 0(0) |
| 2-VD | 20(32.3) | 11(55.0) | 3(30.0) |  | 2(18.18) | 1(14.3) | 0(0) |
| 3-VD | 28(45.1) | 7(35.0) | 5(50.0) |  | 2(18.18) | 0(0) | 1(25.0) |
| Type of affected vessel, no (%) | |  |  |  |  |  |  |
| LM | 3(4.8) | 0(0) | 0(0) |  | 0(0) | 0(0) | 0(0) |
| LAD | 39(62.9) | 19(95.0) | 8(80.0) |  | 6(54.54) | 1(14.3) | 1(25.0) |
| LCX | 26(41.9) | 7(35.0) | 7(70.0) |  | 6(54.54) | 0(0) | 1(25.0) |
| RCA | 34(54.8) | 8(40.0) | 7(70.0) |  | 3(27.27) | 1(14.3) | 1(25.0) |
| Stenosis severity, %, mean, SD) | |  |  |  |  |  |  |
| Overall vessels | 71.6(12.1) | 73.4(13.3) | 81.8(15.1) |  | 51.6(16.4) | 49.44(12.1) | 57.1(30.6) |
| Stent-placed vessels | 92.9(7.52) | 92.4(7.09) | 92.2(6.25) |  | 78.4(10.4) | 87.5(10.6) | 83.3(4.71) |
| Stent-unplaced vessels | 42.5(10.4) | 42.2(11.8) | 45.0(10.0) |  | 40.0(10.5) | 33.6(6.61) | 30.8(5.89) |

PCI: percutaneous coronary intervention; STEMI, ST elevation myocardial infarction; NSTEMI, non-ST-elevation myocardial infarction; UA, unstable angina; SD, standard deviation; 1-VD, single-vessel disease; 2-VD, two-vessel disease; 3-VD, three-vessel disease; N/A, not applicable; LAD, left anterior descending; LCX, left circumflex artery; RCA, right coronary artery.

| **Variables (before PCI)** | **Serum miR-186-5p (before PCI)** | | **Variables (after PCI)** | **Serum miR-186-5p  (after PCI)** | | **Variables ( Δ )** | **ΔSerum miR-186-5p** | |
| --- | --- | --- | --- | --- | --- | --- | --- | --- |
| NT-pro-BNP ^a^ | *r* =-0.007 | *p* =0.953 | NT-pro-BNP ^b^ | *r* =0.123 | *p* =0.482 | ΔNT-pro-BNP ^a^ | *r* =0.381 | *p* =0.055 |
| N/A | N/A | N/A | LVEF ^a^ | *r* =-0.358 | *p* =0.044 | LVEF ^a^ | *r* =0.247 | *p* =0.173 |
| NT-pro-BNP ^c^ | *r* =0.001 | *p* =0.999 | NT-pro-BNP ^c^ | *r* =-0.143 | *p* =0.642 | NT-pro-BNP ^c^ | *r* =-0.002 | *p* =0.998 |
| ΔNT-pro-BNP | *r* =-0.308 | *p* =0.331 | ΔNT-pro-BNP ^b^ | *r* =0.067 | *p* =0.885 | ΔNT-pro-BNP ^b^ | *r* =-0.055 | *p* =0.881 |
| ΔNT-pro-BNP | *r* =-0.190 | *p* =0.651 | ΔNT-pro-BNP ^c^ | *r* =-0.190 | *p* =0.651 | ΔNT-pro-BNP ^c^ | *r* =-0.238 | *p* =0.570 |
| LVEF ^b^ | *r* =0.340 | *p* =0.280 | LVEF ^b^ | *r* =0.134 | *p* =0.731 | LVEF ^b^ | *r* =-0.268 | *p* =0.486 |
| ΔLVEF | *r* =-0.286 | *p* =0.424 | ΔLVEF | *r* =-0.306 | *p* =0.504 | ΔLVEF | *r* =-0.234 | *p* =0.613 |

**Table S3.** Correlations of serum miR-186-5p with LVEF and NT-pro-BNP

NT-pro-BNP^a^, NT-pro-BNP before PCI; NT-pro-BNP^b^, NT-pro-BNP after PCI ; NT-pro-BNP^c^, NT-pro-BNP at follow up; ΔNT-pro-BNP^a^ , NT-pro-BNP before PCI subtract NT-pro-BNP after PCI ; ΔNT-pro-BNP^b^ , NT-pro-BNP before PCI subtract NT-pro-BNP at follow up; ΔNT-pro-BNP^c^ , NT-pro-BNP after PCI subtract NT-pro-BNP at follow up; LVEF^a^ , LVEF after PCI; LVEF^b^, LVEF during follow-up; ΔLVEF, LVEF after PCI subtract LVEF during follow-up
