# Supplementary figures and images for: Association of Serum miR-186-5p With the Prognosis of Acute Coronary Syndrome Patients After Percutaneous Coronary Intervention
Source: Front Physiol. 2019 Jun 5;10:686. doi: 10.3389/fphys.2019.00686 (PMC6560170; doi:10.3389/fphys.2019.00686)

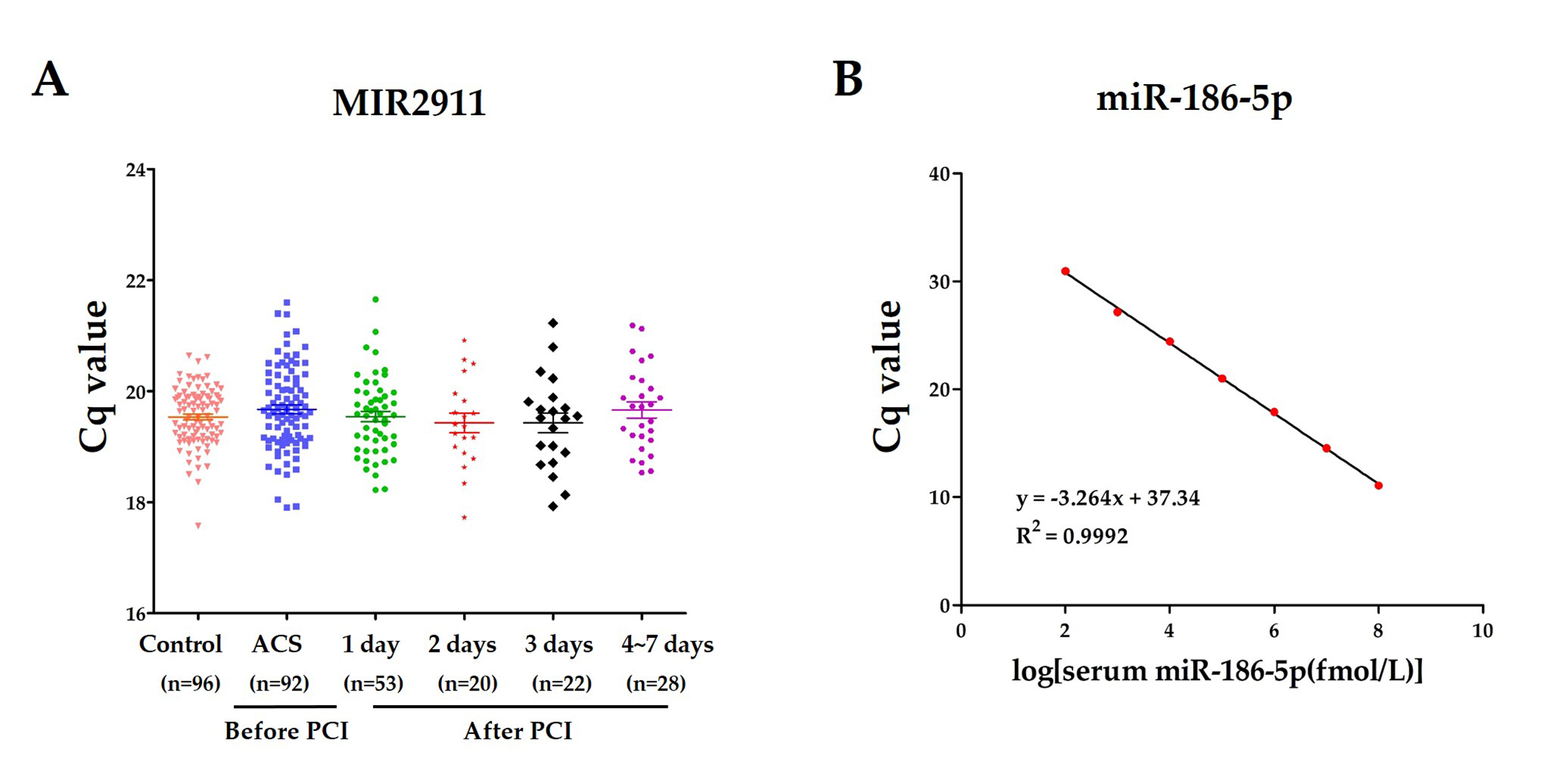

Supplement: Supplementary file 1 [file Data_Sheet_1.zip › Supplementary material/supplementary figures/Figure-S1.tif]

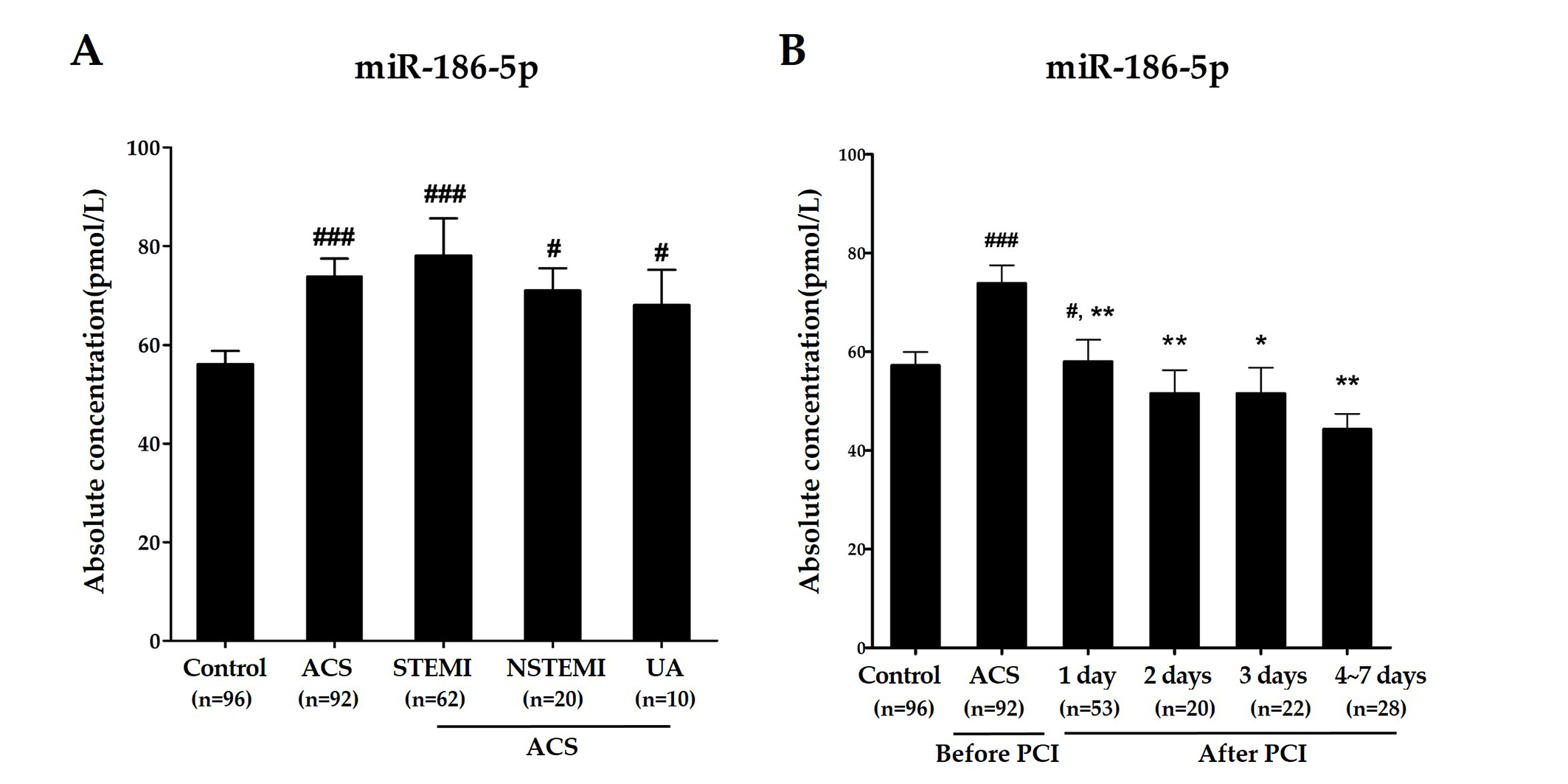

Supplement: Supplementary file 1 [file Data_Sheet_1.zip › Supplementary material/supplementary figures/Figure-S2.tif]

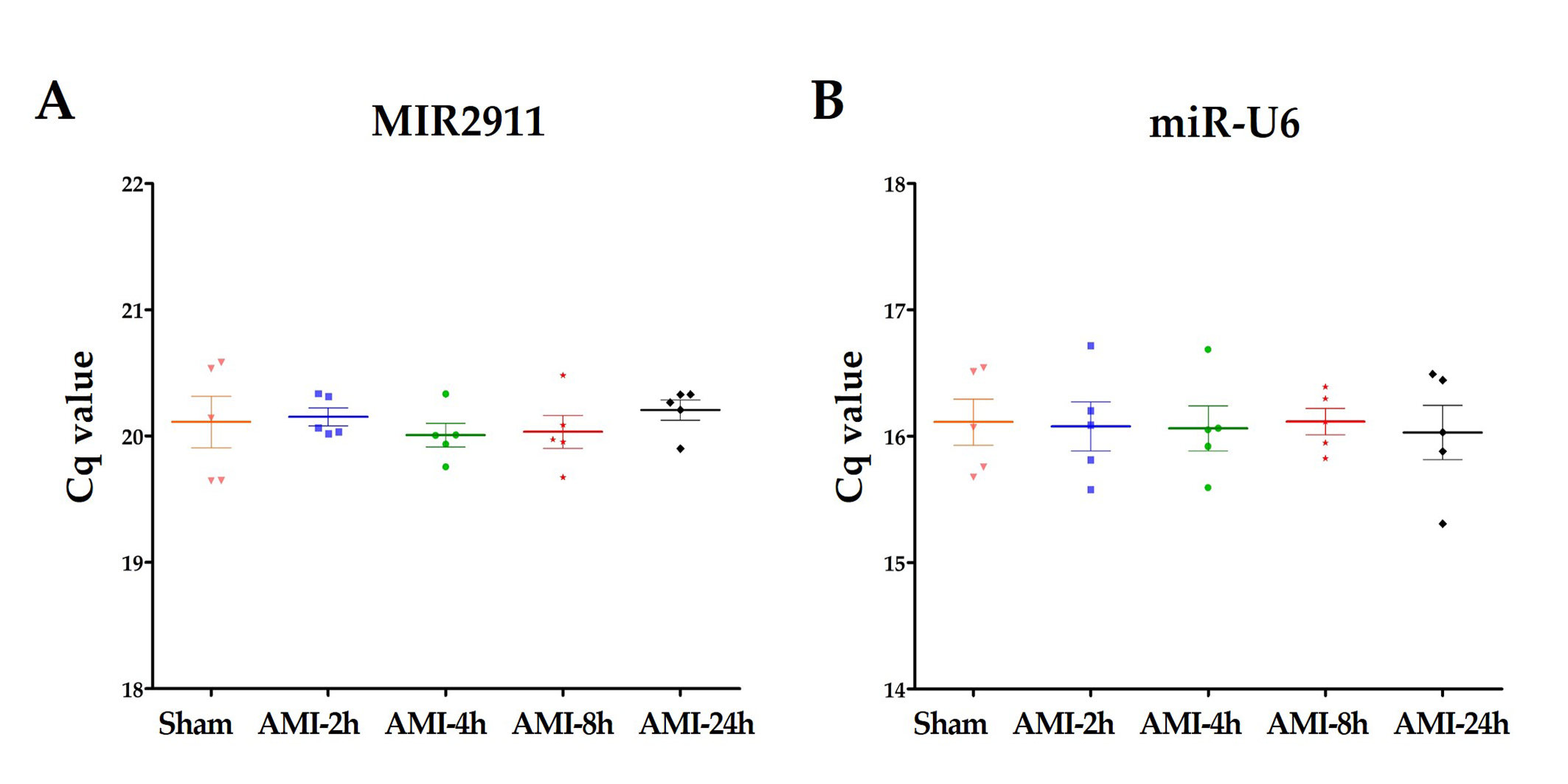

Supplement: Supplementary file 1 [file Data_Sheet_1.zip › Supplementary material/supplementary figures/Figure-S3.tif]

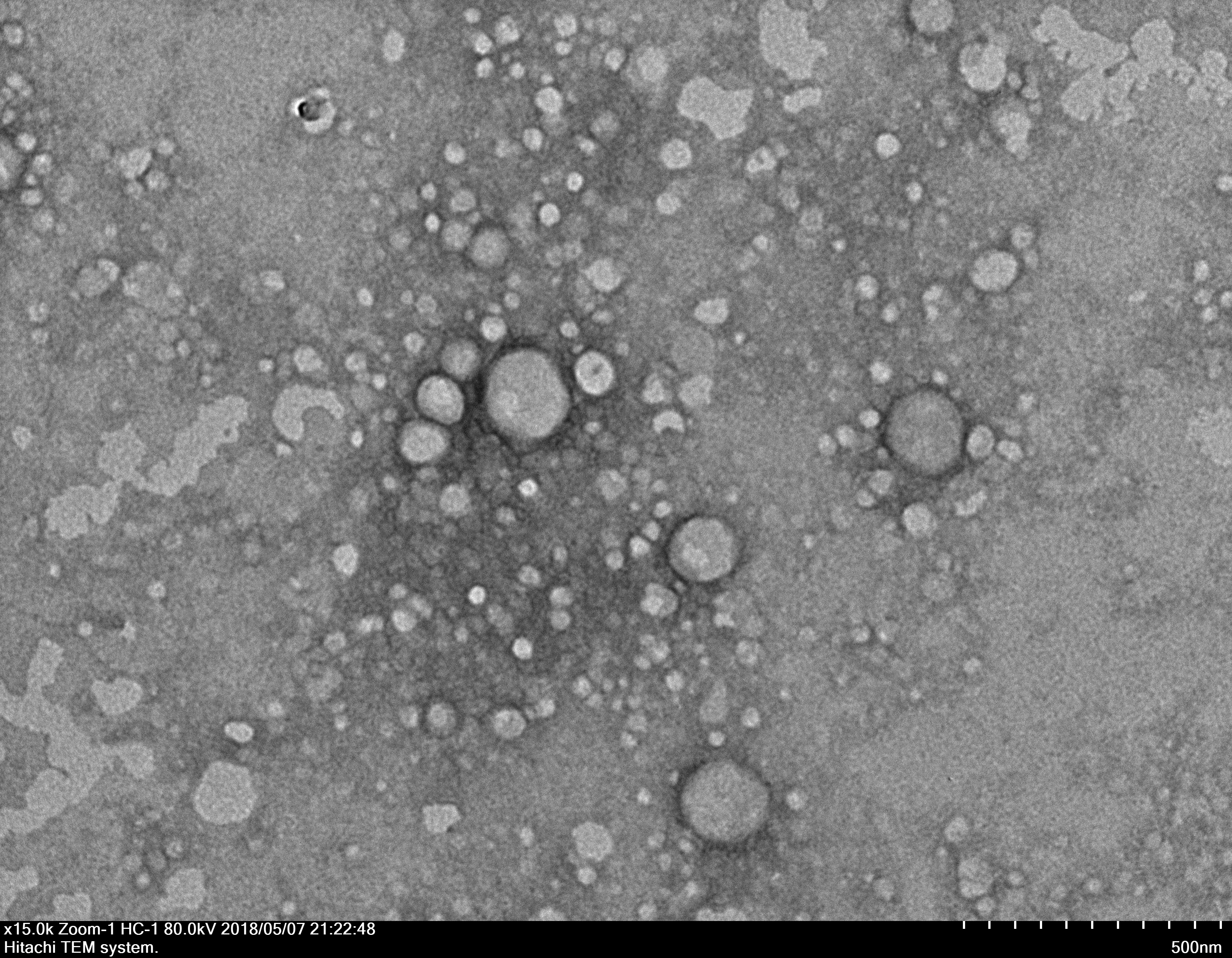

Supplement: Supplementary file 1 [file Data_Sheet_1.zip › Supplementary material/supplementary figures/Figure-S4-original picture/Figure-S4b-TEM.tif]

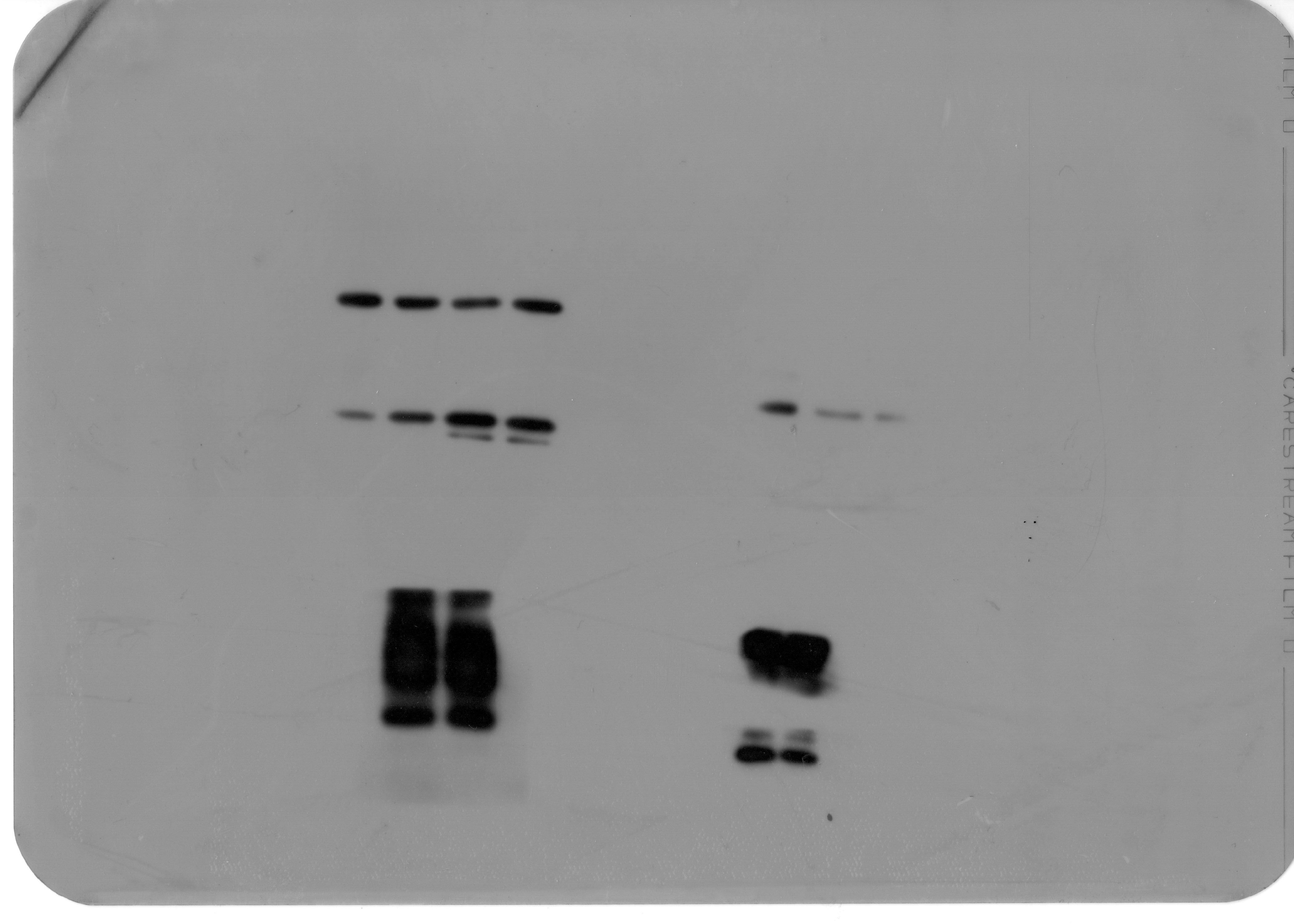

Supplement: Supplementary file 1 [file Data_Sheet_1.zip › Supplementary material/supplementary figures/Figure-S4-original picture/Figure-S4c--WB.jpg]

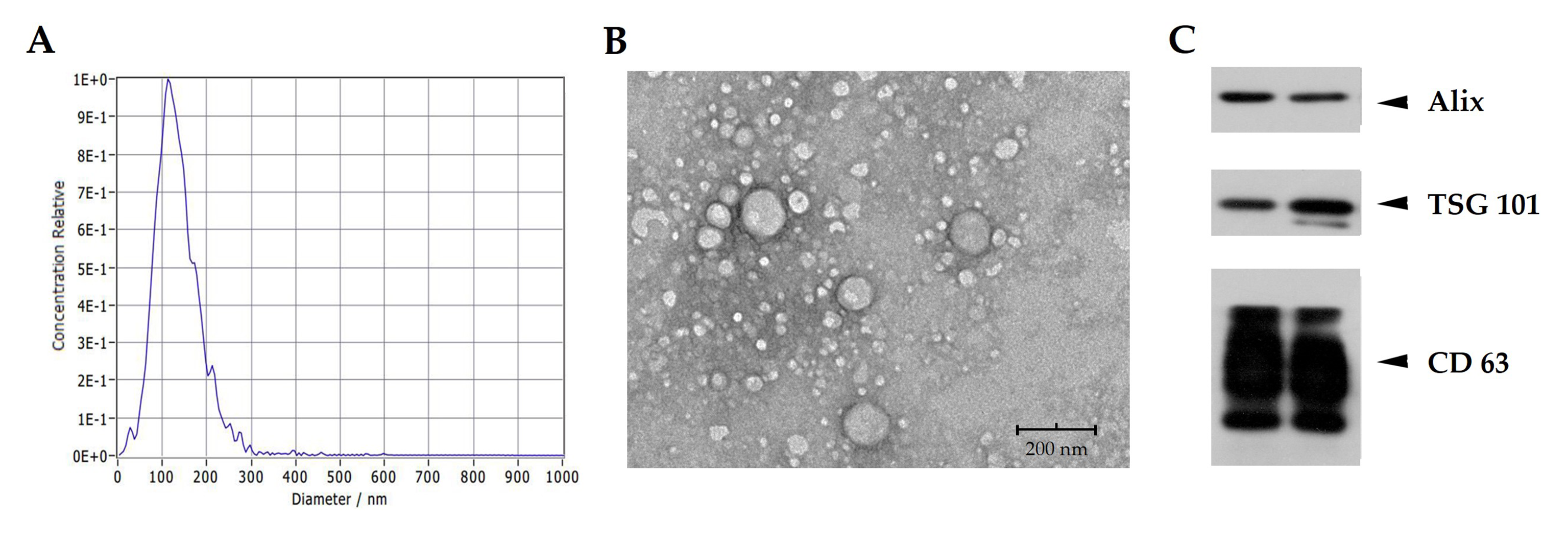

Supplement: Supplementary file 1 [file Data_Sheet_1.zip › Supplementary material/supplementary figures/Figure-S4.tif]

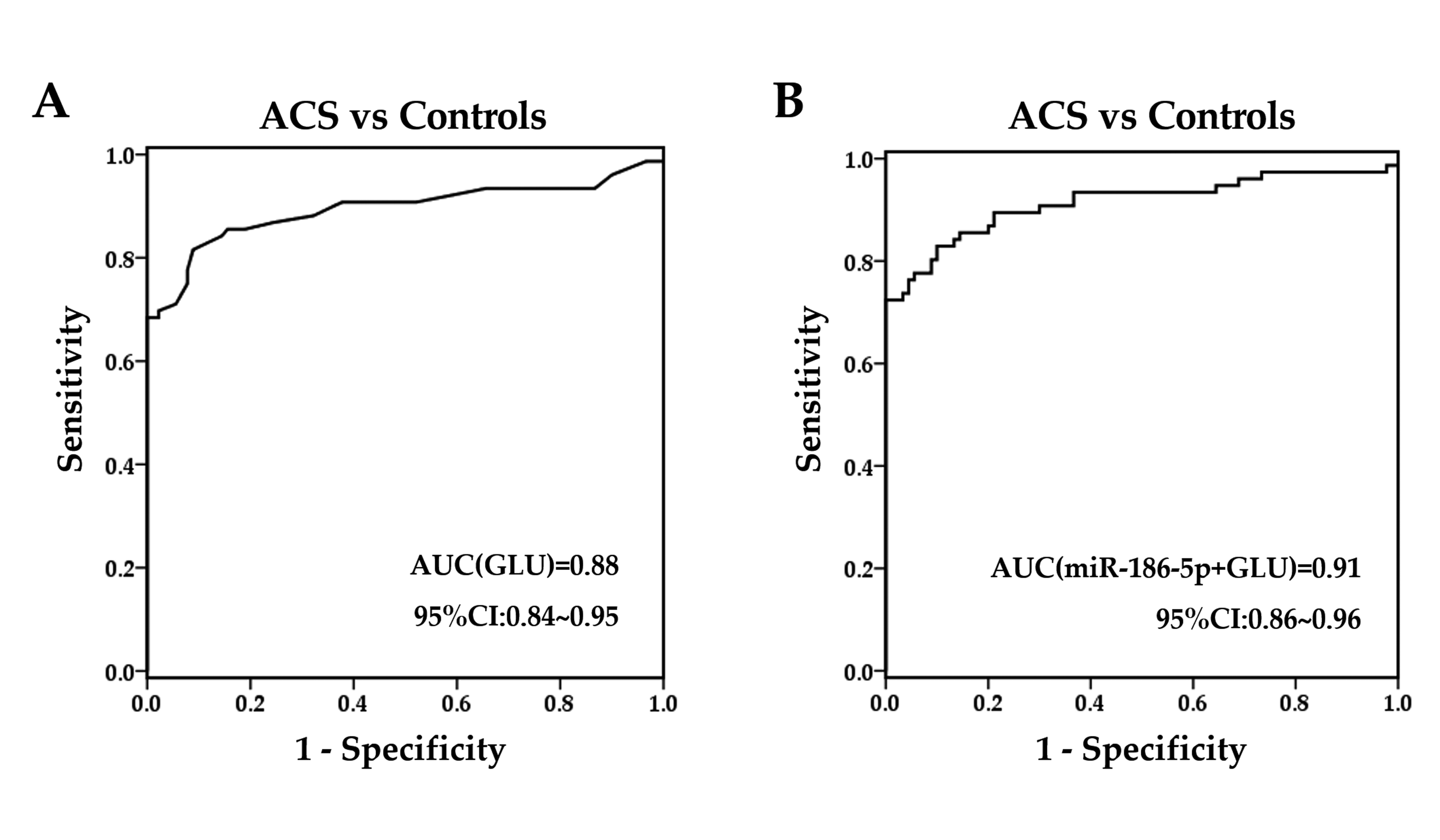

Supplement: Supplementary file 1 [file Data_Sheet_1.zip › Supplementary material/supplementary figures/Figure-S5.tif]
